# The f0 perturbation effects in focus marking: Evidence from Korean and Japanese

**DOI:** 10.1371/journal.pone.0283139

**Published:** 2023-03-23

**Authors:** Si Chen, Yitian Hong, Bei Li, Eunjin Chun

**Affiliations:** 1 Department of Chinese and Bilingual Studies, The Hong Kong Polytechnic University, Hong Kong Special Administrative Region, China; 2 Research Centre for Language, Cognition, and Neuroscience, Department of Chinese and Bilingual Studies, The Hong Kong Polytechnic University, Hong Kong Special Administrative Region, China; 3 Research Institute for Smart Ageing, The Hong Kong Polytechnic University, Hong Kong Special Administrative Region, China; 4 The HK PolyU-PekingU Research Centre on Chinese Linguistics, The Hong Kong Polytechnic University, Hong Kong Special Administrative Region, China; Chulalongkorn University, THAILAND

## Abstract

Many studies showed that prosodic cues such as f0, duration and intensity are used in focus marking cross-linguistically. Usually, on-focus words exhibit expansions of acoustic cues such as f0 expansion, whereas post-focus words may show compression of acoustic cues. However, how features in a sub-syllabic level are employed in focus marking remain to be investigated. F0 perturbation refers to the phenomenon that vocal folds vibration is affected by the preceding non-sonorant consonant. The current study aims to examine how f0 perturbation is realized in focus marking in two languages Japanese and Korean. Tokyo Japanese is a pitch-accent language and Seoul Korean is considered to be at the stage of quasi-tonogenesis. Our results showed that f0 perturbation effects were enhanced in on-focus positions and compressed in pre- and post-focus positions for both narrow and contrastive focus in both languages. In addition, our results showed that pitch accent can also affect the realization of f0 perturbation in various focus conditions. Compared to Korean, our results in Japanese showed that f0 perturbation effects were less restricted. These results provide new insights into the current model of communicative functions that sub-syllabic level acoustic cues such as f0 perturbation can also be employed in focus marking.

## 1. Introduction

In the last few decades, linguistic prominence has been studied cross-linguistically. Linguistic prominence has been defined as the salience of some elements comparing to other surrounding elements. This proposal can be applied to various domains and the prominent unit is usually produced with greater efforts and can be more easily perceived [1]. Moreover, information structure was introduced by [2] and information structure is considered as the formal structure motivated by pragmatic functions [3]. Topic and focus are listed as categories of information structure. Focus is defined as “the semantic component of a pragmatically structured component” [3]. It is possible to use prosodic prominence to mark focus. The prominent unit can be realized via prosodic characteristics to provide listeners with new information (e.g., [1, 4, 5]).

In [3], three types of focus are proposed, including predicate-focus, argument-focus and sentence-focus structure. [6] proposes various types of focus such as broad, narrow and contrastive focus. Broad focus involves a whole sentence, whereas narrow focus usually refers to focus on one constituent [7]. The focus in (1) represents a broad focus and that in (2) is a narrow focus.

1. Broad focus
  - What happened?
  - Emily broke a vase.
2. Narrow focus
  - What did Emily break?
  - Emily broke a vase_._
  Contrastive focus [8] or identificational focus [9], is the focus of a constituent, which usually rejects an alternative such as in (3).
3. Contrastive focus
  - Did Emily break a glass?
  - (No,) Emily broke a vase_._

Previous studies explored the features of f0, intensity and duration in focus marking (e.g., [10, 11]), and yet fewer studies investigated how f0 perturbation effects in the sub-syllabic level may change with respect to focus. F0 perturbation refers to the phenomenon that f0 may be raised or lowered after a non-sonorant consonant (e.g. [12, 13]). Both Korean and Japanese show f0 perturbation effects after stops and they share similar acoustic realizations in focus marking for both on-focus and post-focus targets [14, 15]. The two languages are also different in that Japanese is considered to be either a pitch accent or tonal language [16] while Korean is at the stage of quasi-tonogenesis where the f0 is used to differentiate different consonant types and this f0-stop association has been phonologized [17, 18].

Studies of Chinese languages showed that f0 perturbation effects are more restricted, probably due to the need to preserve tonal contrasts. However, as for f0 perturbation in a pitch accent language such as Tokyo Japanese, studies found that it is not necessary that perturbation effect will be suppressed due to the function of f0 to mark a pitch accent [19]. In Tokyo Japanese, the presence of f0 perturbation effect is more relevant to the word position. It has been found that in word-initial position, f0 and voice onset time (VOT) have been used to distinguish the two-way stops, but f0 is not used in word-middle position. It should be noted that there is some debate about the status of Japanese being a pitch-accent language or being tonal illustrated in Section 1.2.3. Another similar language Kansai Japanese with denser tonal system also showed comparable f0 perturbations to Tokyo Japanese [20].

The relationship between focus marking and f0 perturbation is relatively less investigated. One recent study used a corrective focus task and reported that fortis and aspirated stops raised f0 more than lenis stops to signal focus using words with three syllables [21]. Seoul Korean has been reported to show less difference in VOT between lenis and aspirated stops, but more f0 differences. It is also shown that the decrements of VOT difference and the enhancement of f0 difference were conditioned by the prominence of the syllables [22]. Specifically, they used a corrective focus task and examined disyllables. They found that VOT is still used for the lenis–aspirated distinction in the unfocused condition, and in the focused context, VOT is less used. F0 differences are instead enhanced under focus.

The current study further examines perturbation effects in both monosyllables and disyllables across focus conditions including both narrow and contrastive focus in Korean and Japanese. It aims to test whether cues in the sub-syllabic level, i.e. perturbation effects can be employed to mark focus. The state-of-the-art statistical methods can model and test the f0 contours to show the differences in specific regions for a better comparison across various focus conditions and stop types. It also aims to test how the perturbation effect under focus may show different patterns in these two languages. The following two sections will briefly review the relationship between f0 perturbation effect and tones/pitch accent in the two languages to be examined in the current study.

### 1.1. Prosodic focus marking across languages

In addition to morphosyntactic structures, extensive studies demonstrate prosodic realization of focus, including variations in pitch patterns (e.g., [10]), intensity (e.g., [23]), and duration (e.g., [24]).Focal lengthening and intensity raising of the on-focus components are reported consistently across languages, e.g. English [25], German [26], Korean [14], Japanese [15], Mandarin [27], Hijazi Arabic [4] and indigenous languages such as Yoloxochitl Mixtec [28]. In addition, the pitch of a constituent is found consistently higher when it is under focus in languages such as American English [10]. German [26] also showed a higher f0 peak and Hijazi Arabic showed higher mean f0 values when the on-focused targets were in sentence-initial and sentence-penultimate positions. However, it only showed higher maximum f0 values in sentence-penultimate position [4].

Moreover, Mandarin [29], Shanghainese [30], yoloxóchitl mixtec [28], Japanese [31], Korean [15], Mandarin and English [10, 32] exhibit similar properties of focus, where the pitch range tends to be expanded under focus, compressed in post-focus positions. This phenomenon is referred to as post-focus compression (PFC) [33]. However, PFC is not a universal phenomenon and it is found to be absent in some languages such as Taiwanese, Taiwan Mandarin [11] and Cantonese [34]. The absence and presence of PFC do not seem to relate to whether a language is tonal or non-tonal, or whether morphosyntactic means are available [11], and a cross-linguistic investigation on PFC realization is needed to better understand the underlying mechanism.

Fewer studies examined pre-focus regions, and the findings were less systematic. In two Arabic dialects studies [4], f0 of pre-focus items lacked systematic changes. [35] found the pre-focus compression correlated with syntactic status of the targets, where pre-focus f0 lowering was reported in VP and object foci. On the other hand, [15] suggested that contrastive focus is more likely to trigger pre-focus compression than narrow focus. In addition to on-focus and post-focus positions, the current study also investigates f0 perturbation in pre-focus positions.

### 1.2 F0 perturbation

#### 1.2.1. General findings

F0 perturbation refers to the phenomenon that vocal folds vibration is affected by the preceding non-sonorant consonant. Many early studies reported that f0 rose after a voiceless consonant and decreased after a voiced consonant [12,13,36]. [37] found that f0 rose after voiceless consonants, but it was not lowered after voiced consonants with improved methodology. For true voiced consonants in French and Italian, f0 was lowered only to a marginal significance [38]. The relationship between aspiration of voiceless consonants and f0 perturbation effect is less inconsistent across languages [24].

In addition, the perturbation effects can be constrained by other functions of f0. For example, tonal languages may use f0 for lexical contrasting and a pitch-accent language need to use f0 to mark the pitch accent. Studies of Chinese languages showed that f0 perturbation effects are more restricted, probably due to the need to preserve tonal contrasts. However, as for f0 perturbation in a pitch accent language such as Tokyo Japanese, studies found that it is not necessary that perturbation effect will be suppressed due to the function of f0 to mark a pitch accent [19]. In Tokyo Japanese, the presence of f0 perturbation effect is more relevant to the word position. It has been found that in word-initial position, f0 and VOT have been used to distinguish the two-way stops, but f0 is not used in word-middle position. It should be noted that there is some debate about the status of Japanese being a pitch-accent language or being tonal illustrated in Section 1.2.3. Another similar language Kansai Japanese with denser tonal system also showed comparable f0 perturbations to Tokyo Japanese [20]. The relationship between focus marking and f0 perturbation is relatively less investigated. One recent study used a corrective focus task and reported that fortis and aspirated stops raised f0 more than lenis stops to signal focus using words with three syllables [21]. Seoul Korean has been reported to show less difference in VOT between lenis and aspirated stops, but more f0 differences. It is also shown that the decrements of VOT difference and the enhancement of f0 difference were conditioned by the prominence of the syllables [22]. Specifically, they used a corrective focus task and examined disyllables. They found that VOT is still used for the lenis–aspirated distinction in the unfocused condition, and in the focused context, VOT is less used. F0 differences are instead enhanced under focus. The following two sections will briefly review the relationship between f0 perturbation effect and tones/pitch accent in the two languages to be examined in the current study.

#### 1.2.2. Korean

One of the target languages of our study, Seoul Korean, uses f0 to differentiate consonant types and the f0-stop association has been phonologized in Korean. This sound change is supported by the evidence of an increase in f0 differences and a reduction of VOT differences after lax and aspirated stops both from speech production [17, 18] and perception studies [39]. [18] argued that the current sound change of Korean is at a stage of quasi-tonogenesis, in which the extra pitch feature is being phonologized and becoming distinctive over time [22, 40]. The phonologization of the f0 differences is analyzed by some as phrase-initial prosodic strengthening [41] and a prosodically driven sound change [22]. Specifically, [22] reported a sound change of a reduction of VOT and an enhancement of f0 for the lenis–aspirated stop in domain-initial position and in domain-medial focused position. Korean has a strong microprosodic effect (f0 perturbation) compared to English and French [41], and it might show influence from various focus conditions in pre- post- and on-focus positions.

As for focus realization, similar to most languages reviewed above, Korean is reported to increase its f0 maximum [21] and increase its f0 span [5] for on-focus targets, and it enlarges its difference of mean pitch from the pre-focus item [42].

In addition, inconsistent findings were reported regarding constituents without a focus. [5] found that constituents preceding or following a focused constituent did not show phonetic reduction while [42] reveals a significant difference of mean pitch between pre-focus and on-focus conditions. Post-focused elements were observed with a lower f0 peak [21]. Some of these studies made pairwise comparisons among words in pre-, post- and on-focus conditions, which made it difficult to examine all focus conditions. for example, [21] investigated on-focus vs. post-focus conditions and [43] investigated on-focus vs. pre-focus conditions. The current study sets up a baseline condition for all comparisons.

#### 1.2.3. Japanese

Tokyo Japanese is traditionally considered as a pitch-accent language, which may also be reanalyzed as a tonal language [26]. It bears a tone on each mora that can be associated by rules once the accent marker is known (e.g., [44]). and their tonal realization is more restricted [20].

**Fig 1:**
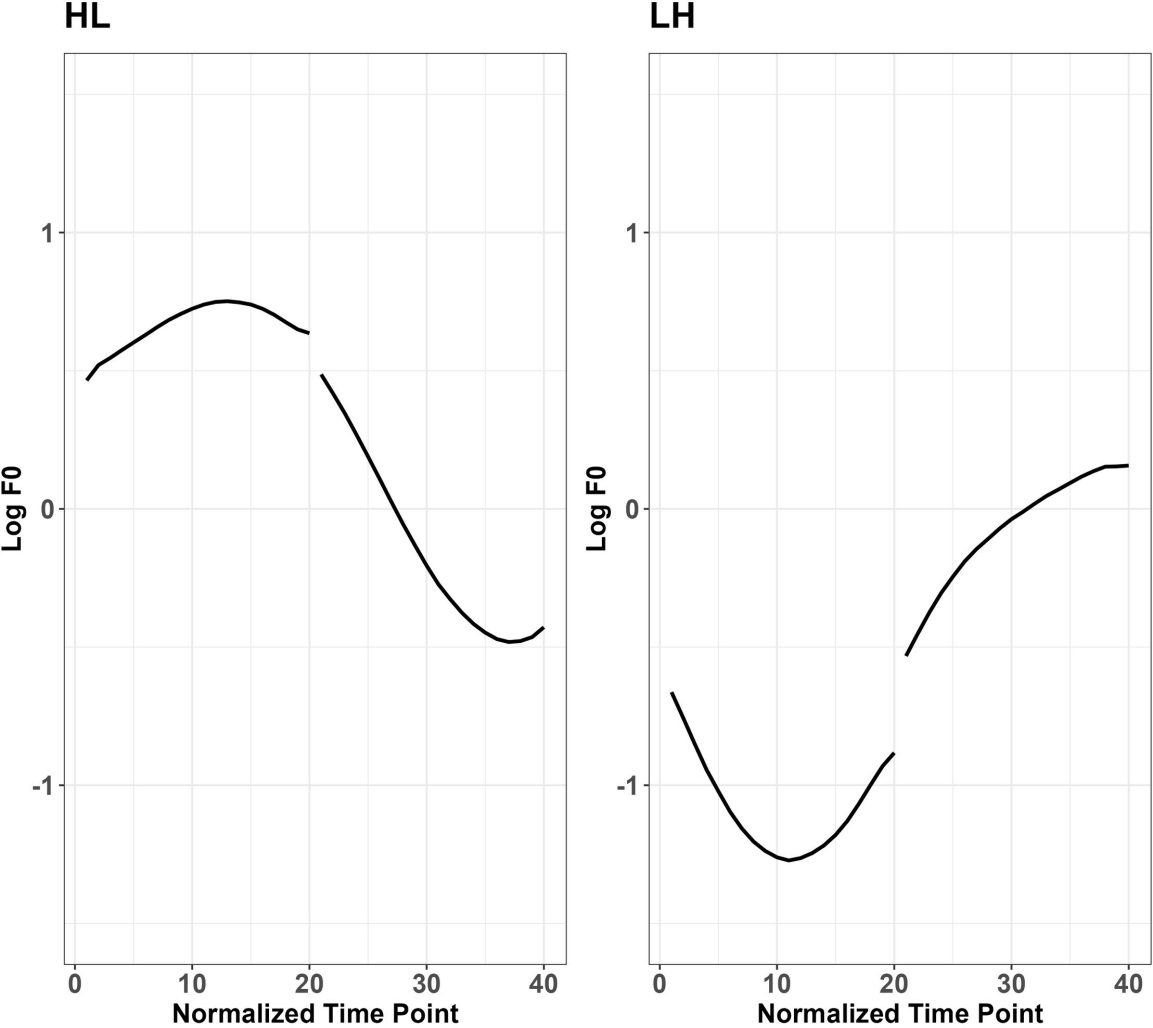
Pitch contours of two disyllabic Japanese words (left: /tako/; right: /taki/) extracted by the baseline production of the current study.

Fig 1 presents examples of two Japanese words where one of them bears HL tone, and the other bears LH tone. Similar to other languages reported in the literature, Japanese shows higher f0 after voiceless consonants than voiced consonants [45–47]. [48] identifies a 40ms dip in f0 after a voiced stop, and in the H-L accent, compared to a voiceless stop, f0 peaks later after a voiced stop due to an initial low f0, whereas in the L-H accent, f0 increases faster following an initial dip after a voiced stop compared to a voiceless stop. [47] shows that although the voiced onset consonant is sometimes devoiced, the perturbation effect observed with a voiced or voiceless onset consonant is consistent. In syllables bearing a pitch accent. [49] found a more salient effect of f0 perturbation in H context than in L context in word initial positions. In order to preserve the pitch accent pattern, the offset of the f0 curves was raised to a higher level in H context while it dropped to a lower level in L context. In [48], voiceless stops were reported to have an earlier f0 peak in HL context and a slower increase of f0 after the drop in LH context, compared with voiced stops. The above studies indicate that f0 perturbation effect tends to be affected by pitch accent in Japanese.

In addition, words under focus were found to exhibit an expansion of f0 range together with an increase of f0 maximum and mean f0 [15]. [50] stated that Japanese shows a boost of the accent peak and PFC for pitch accented words, but f0 patterns were less consistent for unaccented words.

### 1.3 The current study

The current study explored two languages Korean and Japanese to examine how f0 perturbation effect is realized in various focus conditions. The two languages share some similarities in many aspects including morphology, syntax and also the interface between morpho-syntax and prosody. Moreover, accentual phrase (AP) and intonational phrase (IP) are two units within which the prosodic pattern is assigned for both Japanese and Korean [51–54]. Both lengthening of duration and intensity raising of the on-focus components are found and both languages showed post-focus compression [14, 15, 31]. Also, both Korean and Japanese use f0 to indicate a contrast in stop types. The two languages also have some differences. Specifically, since Korean is at a stage of quasi-tonogenesis and Japanese can be analyzed as a pitch accent language, these two languages may need to use f0 to indicate a contrast in stops or to mark an accent in addition to the focal function. These similarities and differences in focus marking and f0 perturbation provide us with an opportunity to understand the interaction between f0 perturbation and focus marking.

The current study aims to answer the following research questions: 1) Are f0 perturbation effects magnified under focus for both narrow and contrastive focus in Korean and Japanese? 2) Are perturbation effects compressed on constituents without focus? 3) How do perturbation effects play a role in different languages?

Regarding the first research question, this study aims to investigate the role of perturbation effects in focus marking. Although extensive studies showed changes of f0, intensity and duration in focus marking cross-linguistically (e.g., [10, 11, 24, 31]), relatively less is known about how cues within a syllable might be used in focus marking. The current study examines monosyllables and disyllables with different types of onset stops in Korean and Japanese. In on-focus targets, acoustic cues such as f0 range and duration are usually magnified to make the targets more salient, therefore it is likely that for both languages, perturbation effects are also magnified for on-focus items. For Korean, f0 is used to differentiate stop types, and f0 contrasts across stop types might also be enhanced under focus. For Japanese, since it was reported that perturbation effect was not suppressed to a large extent due to the function of marking a pitch accent [19], it is likely that under focus, perturbation effects will not be suppressed either. In addition, since Tokyo Japanese also uses f0 to mark pitch accent, the realization of f0 perturbation might be further affected by different pitch accents.

As for the second research question, the phenomenon of PFC (e.g., [11, 33, 34]) was reported cross-linguistically. However, little is known about how acoustic cues within a syllable plays a role in syllables before and after the focused target in Korean and Japanese. Korean and Japanese have been reported to show PFC and it is likely that for both languages, perturbations effects will be suppressed on post-focus syllables.

For the third research question, communicative functions f0 can be used to encode such as lexical meaning, focus and emotions. Tonal languages tend to suppress f0 changes for other functions such as the focal function, which may be due to the necessity of maintaining the pitch contours for lexical contrasts [55]. In addition to focus, Korean and Japanese employs f0 for other purposes. Korean uses f0 to differentiate onset stops, and Japanese used f0 in marking a pitch accent. These two languages may thus show differences in the degree of perturbation effects across focus conditions due to different functions of f0 needed in addition to focus marking.

## 2. Materials and methods

### 2.1 Subjects

Nineteen Seoul Korean native speakers (nine male speakers, ten female speakers; mean age = 26.4 ± 9.0) and thirteen Tokyo Japanese native speakers participated in the experiments of focus production (nine female speakers, six male speakers; mean age = 28.9 ± 9.2). None of the participants reported any history of speaking, hearing, or language difficulties. An overview of the participant information can be found in Appendix IIA of S1 Appendix. The recording of one Korean female speaker (F13) was excluded because she mispronounced many target words and her pronunciation was unclear overall. Two male Korean speakers did not finish the recording of the stimuli with the vowels [e] and [o]. Therefore, they only contributed to the data of the vowel [a] targets.

### 2.2. Materials

#### 2.2.1 Korean

Both monosyllabic and disyllabic targets were designed in the experiment. Korean is a language with a three-way laryngeal contrast of voiceless stops: aspirated stops ([p^h^, t^h^, k^h^]), fortis stops ([p', t', k']) and lenis stops ([p, t, k]) [14, 17]. These nine voiceless stops were selected to combine with three vowels (i.e., [a], [o], [e]) to form 27 consonant-vowel (CV) monosyllabic targets. For disyllabic targets, the C1V1C2V2 syllable was used, in which C1 was [p^h^, t^h^, k^h^, p, t, k, p', t', k'], V1 was controlled as [a] while C2 was controlled as nasal [n] or liquid [l], V2 was controlled as [e], [i], [u].

Each target syllable was recorded in seven focus conditions with two repetitions in each condition. The seven conditions include a baseline condition and six focus conditions. Participants produced the same sentences in all the conditions. In the baseline condition, no question was designed for elicitation of focus. The subject produced the target sentences directly.

In focus conditions, questions were designed to elicit focus in the answers. The target syllables are supposed to be produced in the pre-focus, on-focus and post-focus position based on the elicited questions. Examples of the conditions were shown in Table 1. All the designed questions were recorded by a female Korean native speaker aged twenty-two, who had not obtained any musical training in the past five years. Her self-reported language background is shown in Appendix IIB1 of S1 Appendix. In total, each subject produced 504 sentences ((27 monosyllabic targets + 9 disyllabic targets) * 7 discourse conditions * 2 repetitions).

**Table 1:** Examples of Korean target syllables in various discourse conditions (focused parts are highlighted underlined and in bold).

Question	Gloss	Discourse condition	Target answer (focused part)	Gloss (focused part)
\	**\**	Baseline	민수가 ‘바’를 먹고 있습니다	Minsu is eating ‘pa’.
민수가 ‘바’를 어떻게 하고 있습니까?	What is Minsu doing with ‘pa’?	Narrow pre-focus	민수가 ‘바’를 먹고 있습니다	Minsu is eating ‘pa’.
민수가 무엇을 먹고 있는 것 입니까?	What is Minsu eating?	Narrow on-focus	민수가 ‘바’를 먹고 있습니다	Minsu is eating ‘pa’.
누가 ‘바’를 먹고 있습니까?	Who is eating ‘pa’?	Narrow post-focus	민수가 ‘바’를 먹고 있습니다	Minsu is eating ‘pa’.
민수가 ‘바’를 씻고 있습니까?	Is Minsu washing ‘pa’?	Contrastive pre-focus	민수가 ‘바’를 먹고 있습니다	Minsu is eating ‘pa’.
민수가 사과를 먹고 있습니까?	Is Minsu eating an apple?	Contrastive on-focus	민수가 ‘바’를 먹고 있습니다	Minsu is eating ‘pa’.

#### 2.2.2 Japanese

Both monosyllabic and disyllabic targets were designed and recorded. Monosyllabic targets were CV syllables where C = [pʰ, tʰ, kʰ, b, d, g] and V = [a, o, e]. For disyllabic targets, the C1V1C2V2 syllable was used, where C1 was controlled to be [tʰ] or [d] to maximize the pairs with LH and HL pitch accent, V1 = [a, o, e], C2 was controlled to be [k] and V1 = [i, u, e]. Since Japanese is a pitch-accent language [56, 57], disyllabic targets were also created to bear low-high (LH) or high-low (HL) accent patterns. Therefore, 30 targets were created in total (6*3 monosyllables + 2*3*2 disyllables).

Similar to Korean stimuli, sentences containing the monosyllabic and disyllabic targets with seven different discourse conditions were recorded twice. The baseline condition is designed to be a sentence with the same words as the answers in focus conditions, but without a question to elicit focus. For focus conditions, they answered a pre-recorded question. Table 2 showed the examples of discourse conditions.

**Table 2:** Examples of Japanese target syllables in various discourse conditions (focused parts are highlighted underlined and in bold).

Question	Gloss	Discourse condition	Target answer (focused part)	Gloss (focused part)
\	**\**	Baseline	ゆいは「ぱ」を食べています。	Yui is eating ‘pa’.
ゆいは「ぱ」で何をしていますか?	What is Yui doing with ‘pa’?	Narrow pre-focus	ゆいは「ぱ」を食べています。	Yui is eating ‘pa’.
ゆいは何を食べていますか?	What is Yui eating?	Narrow on-focus	ゆいは「ぱ」を食べています。	Yui is eating ‘pa’.
誰が「ぱ」を食べていますか?	Who is eating ‘pa’?	Narrow post-focus	ゆいは「ぱ」を食べています。	Yui is eating ‘pa’.
ゆいは「ぱ」を洗っていますか?	Is Yui washing ‘pa’?	Contrastive pre-focus	ゆいは「ぱ」を食べています。	Yui is eating ‘pa’.
ゆいはりんごを食べていますか?	Is Yui eating an apple?	Contrastive on-focus	ゆいは「ぱ」を食べています。	Yui is eating ‘pa’.

In total, each subject produced by 420 sentences (30 targets * 7 discourse conditions * 2 repetitions). Elicited questions (see Appendix IIB in S1 Appendix) were recorded by a female native Japanese speaker aged thirty-five. She has not obtained any musical training in the past five years. Her self-reported language background is shown in Appendix IIB2 of S1 Appendix.

### 2.3 Procedure

Both Korean and Japanese participants were recorded at a sound-proof booth at the speech lab of the Hong Kong Polytechnic University with a computer and an Azden ECZ- 990 microphone. Participants were recorded using the software Audacity on the computer via the microphone with a sampling rate of 44.1kHz. All participants were paid for their participation and we obtained their written consent in compliance with a protocol approved by the Human Subjects Ethics Sub-committee at the Hong Kong Polytechnic University.

The software E-prime [58] was designed to present participants with randomized stimuli containing pre-recorded questions in Korean and Japanese to elicit speech production of answers with various focus conditions. The program was designed to be self-paced and the participants were instructed to answer the questions with the target sentences every time they heard a question. Before the experimental sessions, the participants familiarized themselves with the stimuli and they also practiced the tasks in a practice session. In the first experimental session, the baseline sentences were presented on a computer screen in a randomized order and participants were asked to produce the sentences. In the second experimental session, pre-recorded questions eliciting focus were played to them and both the questions and the corresponding answers on the screen were shown to them on a computer screen. All the questions were played twice in total. The program was designed to be self-paced, and the participants were instructed to answer the questions as if they are engaged in a natural conversation.

Each target syllable was recorded in seven focus conditions. The participant was asked to treat the target word as the name of a new kind of fruit. The seven conditions include a baseline condition and six focus conditions. They produced the same sentences in all the conditions. In the baseline condition, no question was designed for elicitation of focus. In focus conditions, questions were designed to elicit focus in the answers (see Appendix IA and IIB in S1 Appendix). The monosyllabic targets and disyllabic targets were elicited with two repetitions in each condition.

### 2.4 Data analysis

#### 2.4.1 Extraction of f0 and normalization

The vowels were segmented manually using Praat [59], following the segmentation procedure described by [60]. The f0 values were then extracted using the Praat script ProsodyPro [61] at 20 normalized time points in each segmented portion. Log z-score transformation as shown by [62] was applied to normalize f0 values so as to better compare the results across different individuals.

#### 2.4.2 Functional data analysis

Functional data analysis was applied to compare the difference between pairs of f0 contours and the significantly different regions were reported between each pair as introduced by [55].

Specifically, pairs of normalized f0 contours were first modelled using the following model:

$$y_{i}(t_{j})=f_{i}(t_{j})+\varepsilon_{ij}$$ (1)

where *y*_*i*_(*t*_*j*_) represents the f0 value (after normalization) at time point *t_j_* in the contour *i* produced by the participant and *i* = 1, …,*n* and *j* = 1, …, *m*. The term *ε_ij_* is the error term that has a normal distribution N(0, σ^2^). In order to model each pair of f0 contours, twenty break points were chosen, and four B-spline basis functions were used to fit the curves. A basis function expansion for f_i_(t_j_) was used to fit discrete observations in the form of the following.

$$f_{i}(t)=\;\sum_{k=1}^{k}C_{ki}\;\varphi_{k}(t)$$

where C_ki_ is the coefficient for the k^th^ basis function used to model the i^th^ utterance. For each pair of f0 contours, 20 break points were chosen and four B-spline basis functions were used to fit f0 curves. The optimal smoothing values, λ, were determined by the generalized cross-validation measure (GCV).

Then functional t-tests were conducted to test whether there were regions of significant differences between any pair of f0 contours. The test statistic is the maximum of the multivariate t-test T(t) based on a permutation test (R command: tperm.fd()). A functional t-test constructs a null distribution by shuffling the labels of the two curves randomly and recalculate the maximum of T(t) with the new labels. The formula of T(t) is given below. For each pair of comparison, 200 random samples were used, and calculated observed t-statistic, point-wise 0.05 critical value and maximum 0.05 critical value. When observed t-statistics exceed maximum 0.05 critical values, statistical significance is reached. When observed t-statistics exceed point-wise 0.05 critical value, marginal significance is reached. Details of the model can be found in [63, 64]. A Bonferroni correction for multiple comparisons was used. The critical value is calculated based on the corrected alpha value. For simplicity reasons, only several examples with all the observed t-statistics and critical values were reported in the S1 Appendix. In the results section, only the significant regions were reported to avoid reporting hundreds of observed t-statistics. The details of the modelling procedure can be found in [55].


$$T(t)=\;\frac{\left|\bar{x_{1}}(t)-\bar{x_{2}}(t\right)}{\sqrt{\frac{1}{n_{1}}Var\;[x_{1}(t)]+\frac{1}{n_{2}}Var\;[x_{2}(t)]}}$$

In this study, two types of comparisons were made: (1) pairwise comparisons of f0 contours were conducted on the baseline vs. six focus (narrow and contrastive pre-, post- and on-focus) conditions, respectively; (2) the f0 differences across various stop types were compared (i.e., the differences among lenis, fortis and aspirated stops for Korean and the differences between voiced and voiceless stops for Japanese) in the baseline and six focus conditions respectively. Monosyllables and the first syllables of disyllables were modeled and compared respectively. All these analyses were carried out by R [65]. The R package “fda” verision 6.0.5 was used [63, 64].

#### 2.4.3 Magnitude of f0 perturbation

In order to investigate the degree of perturbation across different focus conditions, the mean f0 of each repetition was extracted and an averaged mean f0 in each target for each subject and discourse condition was calculated (e.g., the averaged mean f0 of Korean /pa/ in on-focus condition for subject M1). Then we calculated the magnitude of mean f0 change by subtracting the mean f0 in baseline from the mean f0 in focus condition, yielding FBDiff (FBDiff = mean f0 of the same target in focus conditions–mean f0 of the same target in baseline). Therefore, six FBDiff were obtained for each target per person. Linear mixed effects models were fitted using the ‘lmerTest’ package [66] in R [65] to determine whether the magnitude of f0 perturbation was affected by stop types and focus conditions.

## 3. Results

### 3.1 Comparisons across focus conditions

#### 3.1.1 Korean

**Fig 2:**
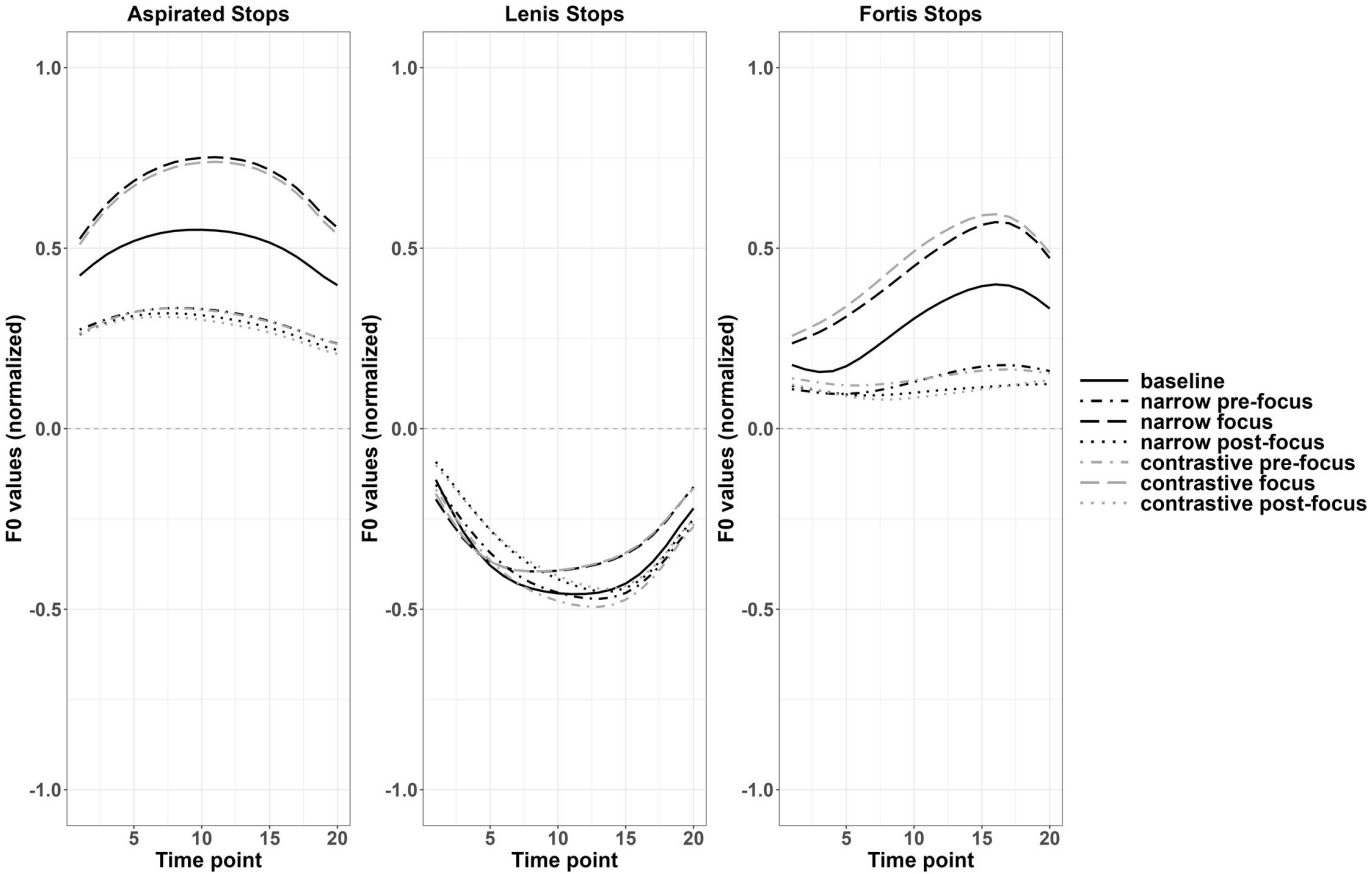
Normalized f0 contours of monosyllabic targets across various discourse conditions (Korean).

We compared changes of f0 contours among seven discourse conditions: baseline (a statement without focus), narrow focus (pre-focus, on-focus, and post-focus), and contrastive focus (pre-focus, on-focus, and post-focus). For Korean data, no differences of f0 contours after three types of stops between focused (i.e., pre-focus, on-focus, post-focus) conditions and the baseline in f0 contours reached significance. As shown in Fig 2, lenis stops remain almost unchanged except for the slightly higher f0 values starting from the first one third of the contours in narrow on-focus and contrastive on-focus conditions. For fortis stops and aspirated stops, the overall f0 values increased in two on-focus conditions, comparing to the baseline and the f0 values reduced in general in all the pre-focus and post-focus conditions, which indicates that Korean speakers tended to increase their f0 for on-focus targets but lower f0 when there is a focus before or after the targets, though the differences did not reach significance. The increase of f0 values for on-focus targets occurred from the initial part of the contour. Moreover, for fortis stops, the f0 curves for pre-focus and post-focus targets were much flatter than the on-focus targets and the baseline, reducing the variation of f0 values. Since the observed t-test statistics for each pair of comparison have around 100 values, only the significant region where the observed t-test statistics exceeded the critical value was reported. An example of the comparison between contrastive pre-focus and the baseline for vowels after fortis stops in monosyllables and disyllables with observed t-values and the critical values are reported with a Fig A1 in Appendix III of S1 Appendix.

**Fig 3:**
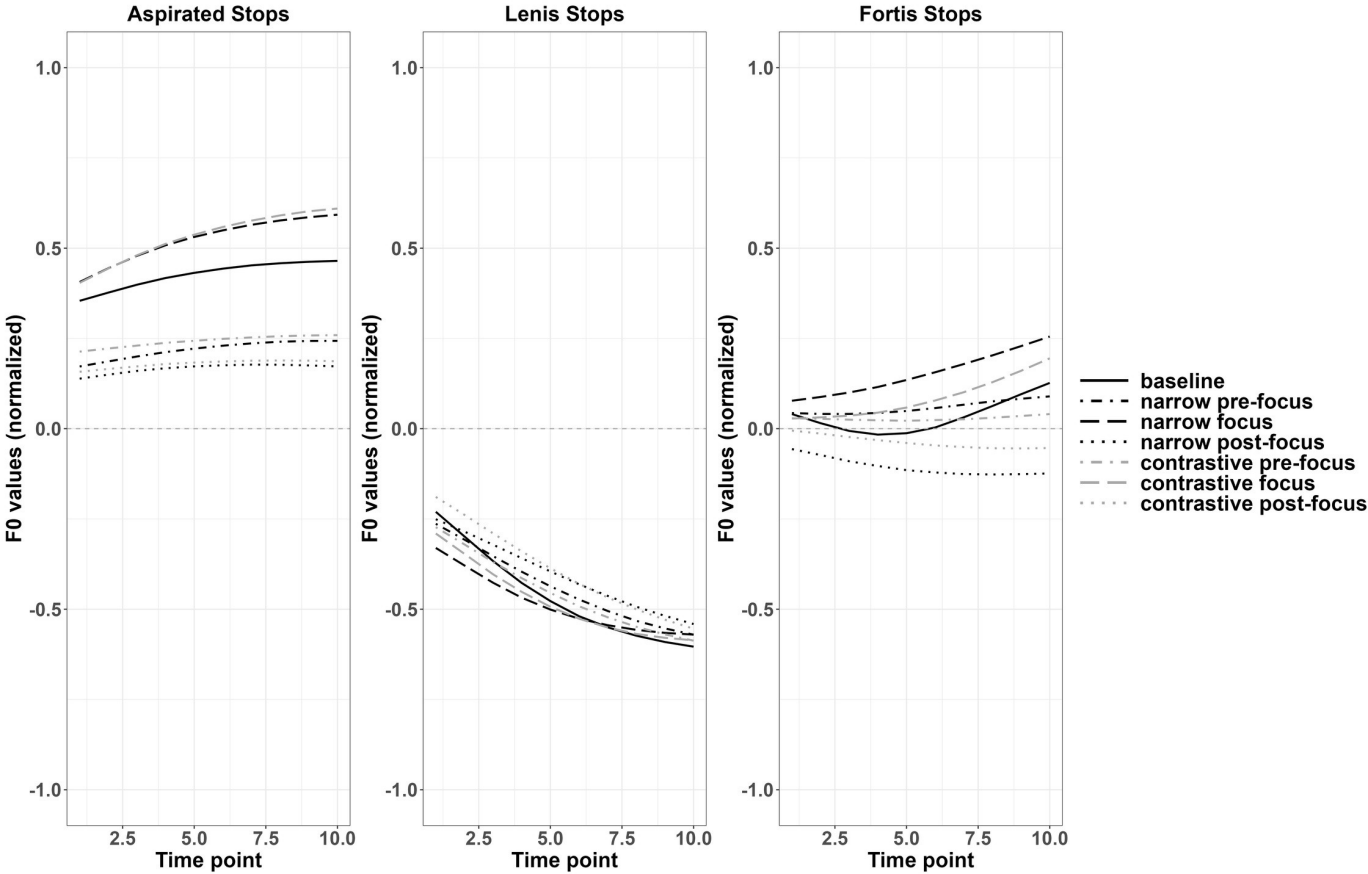
Normalized f0 contours of disyllabic targets in different discourse conditions (Korean).

For the first syllable of Korean disyllabic targets, similar to the findings in monosyllables, no significant differences were found across focus conditions for f0 contours after these three types of stops. However, from Fig 3, a slight increase in f0 values was observed toward the middle of the f0 contours for the two on-focus conditions (narrow and contrastive on-focus conditions) and the f0 values further increased toward the end of the contours especially for aspirated and fortis stops.

#### 3.1.2 Japanese

**Fig 4:**
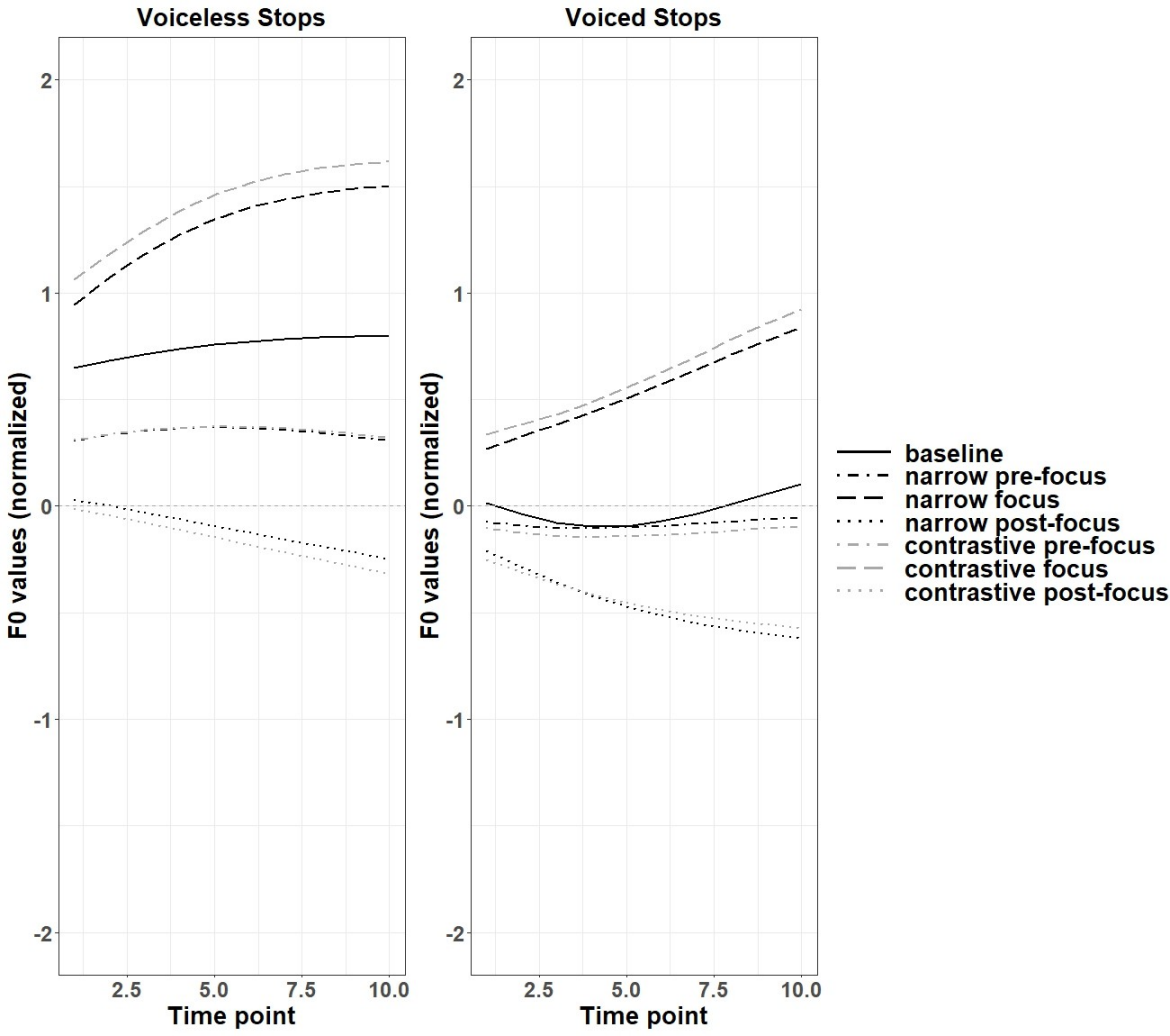
Normalized f0 contours of monosyllabic targets across various discourse conditions (Japanese).

For Japanese monosyllabic targets, significant differences were found in f0 contours between various focus conditions and the baseline for voiceless stops. The two f0 contours in the pairs were significantly different from the beginning to the end. For voiced stops, the significant regions were smaller in two pre-focus conditions compared to the baseline, as shown in Table 3. Referring to the f0 contours shown in Fig 3, for voiceless stops, Japanese speakers raised the values in the whole f0 contour of the target words under focus and suppressed them when there was a focus before or after it. The same changes were found for f0 contours after voiced stops, but for the initial part of the f0 contours in the two pre-focus conditions (narrow and contrastive focus), their differences from the baseline were not significant. An example of the comparison between narrow on-focus and the baseline for f0 contours after voiced stops in monosyllables and f0 contours after voiced and voiceless stops in HL disyllables with observed t-values and the critical values are reported with Fig A2 in Appendix III of S1 Appendix. From the statistical results and Fig 4, Japanese speakers adjusted f0 values to a greater extent in each focus condition compared to Korean speakers, especially in the initial part of f0 contours, indicating a larger perturbation effect.

**Table 3:** Significantly different regions across six focus conditions and the baseline for Japanese monosyllabic targets based on functional t-tests (Marginally significant differences are marked in parentheses).

Pairs	Narrow pre-focus vs. Base	Narrow on-focus vs. Base	Narrow post-focus vs. Base	Contrastive pre-focus vs. Base	Contrastive on-focus vs. Base	Contrastive post-focus vs. Base
**Voiceless stop**	0~100%	2.97%~100% (0~2.97%)	0~100%	0~100%	0~100%	0~100%
**Voiced stop**	72.28%~100% (57.43%~72.28%)	0%∼100%	6.93%~100% (0~6.93%)	51.49%~100% (49.5%~51.49%)	0∼100%	0∼100%

**Fig 5:**
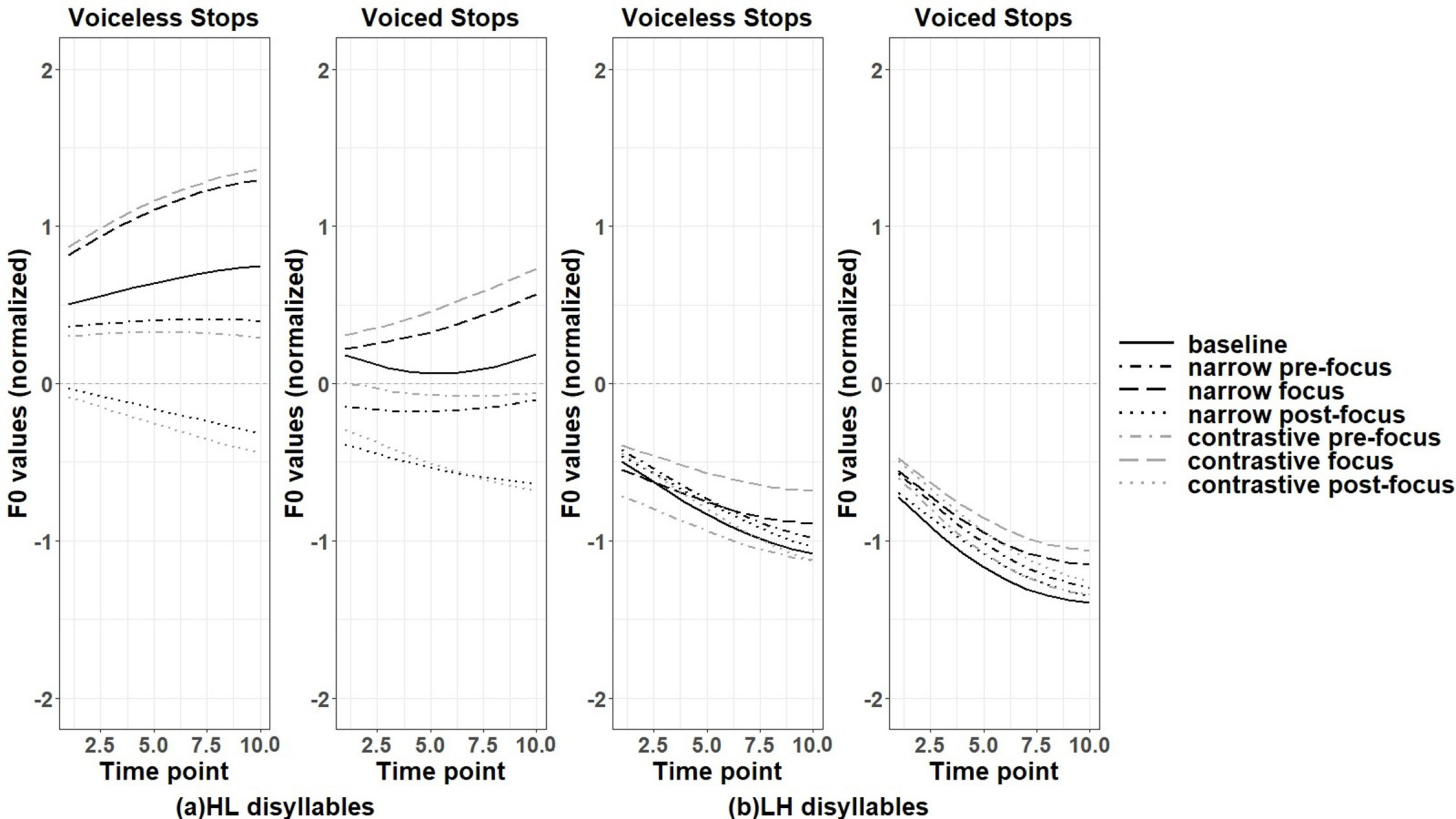
Normalized f0 contours of the first syllable of disyllabic targets in various discourse conditions (Japanese).

**Fig 6:**
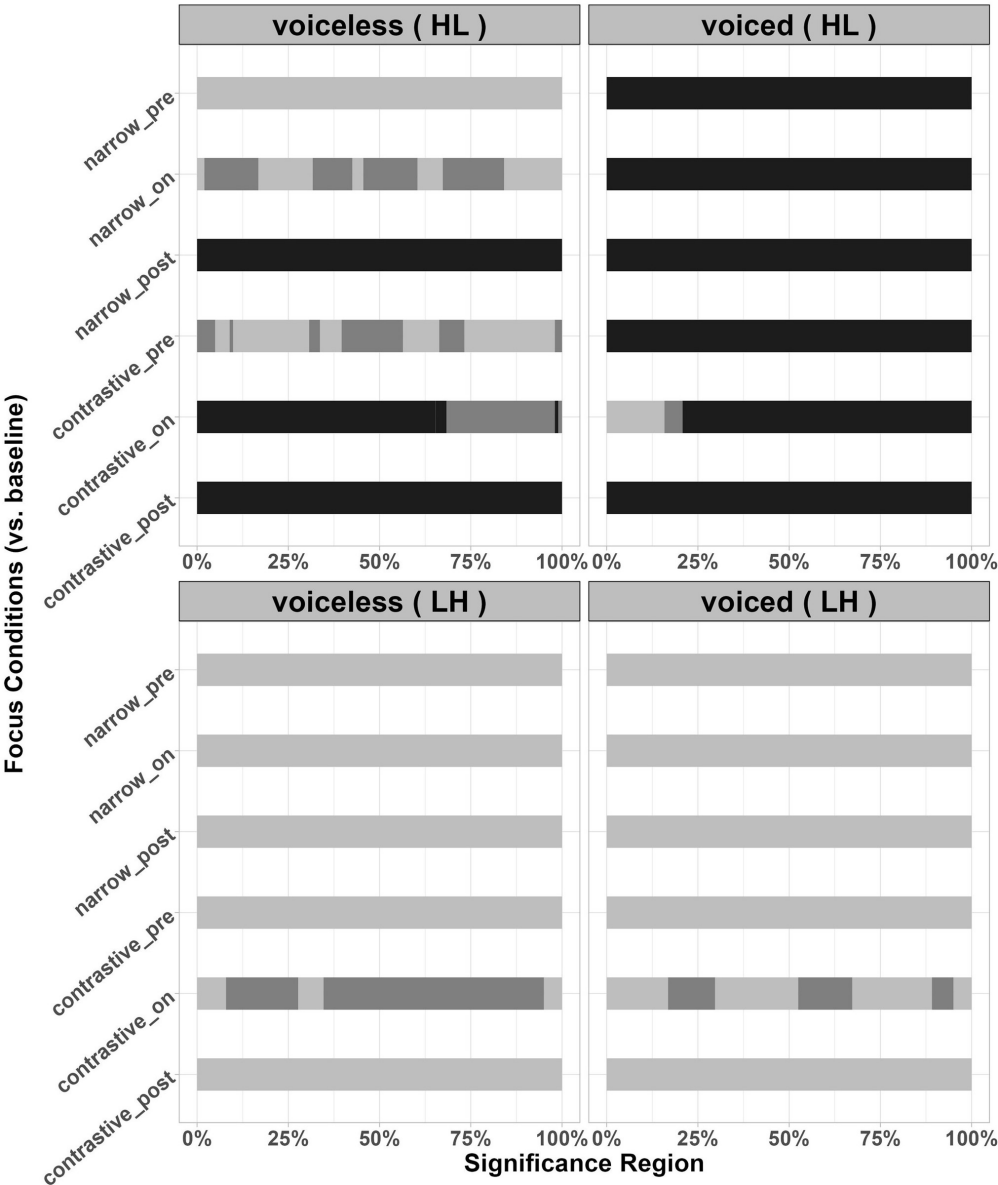
Significant regions between focused conditions and the baseline in disyllabic targets (the darkest part of the bar indicates regions of significant differences; the dark grey part indicates regions of marginal significance; the light grey part signals regions of no significant differences; the labels of y axis indicate various focus conditions compared to the baseline, n = narrow focus, c = contrastive focus).

For Japanese disyllabic targets, two pitch accent patterns were examined: High-Low (HL) and Low-High (LH) pitch accent targets respectively. For the pitch accent pattern HL, changes of f0 contours were similar to Japanese monosyllabic targets, where f0 values were raised in on-focus conditions and were lowered in pre- and post-focus conditions, compared to the baseline. Particularly, f0 values were lowered more in post-focus conditions than in pre-focus conditions, as shown in Fig 5A. The results of functional t-tests in Table VA in the S1 Appendix are consistent with this observation. Fig 6 better illustrates the significance, where the black part indicates the region of statistically significant differences, the dark grey part indicates marginally significant differences while the light grey part signals no significant difference. The statistics details related to Fig 6 can be found in Appendix V of S1 Appendix.

For voiceless stops in HL disyllables, marginally significant differences were found in contrastive pre-focus conditions while in on-focus conditions, the significant regions, marginally significant regions and non-significant regions were shown alternately. For contrastive on-focus condition, the initial part of the f0 contour was significantly higher than the baseline while for narrow on-focus condition, the initial part of f0 contour was higher with marginal significance. The entire f0 contours reached significant differences between post-focus conditions and baselines. For voiced stops, the differences between pre/on/post-focus vs. baseline all reached significance for the entire f0 contours except for the contrastive on-focus condition.

For disyllabic LH targets, according to Fig 5B, all the f0 contours raised slightly in the entire contour compared to the baseline, except for the contrastive pre-focus voiceless targets. However, unlike HL targets, only contrastive on-focus condition showed marginally significant increase of f0 values for both stops, indicating less perturbation effects in LH targets. The f0 values of post-focus targets tended to drop in the second half of the contours. As presented in Fig 6, a marginal significance and non-significance alternated pattern was observed in contrastive on-focus vs. baseline comparisons in both voiced and voiceless LH targets. The statistics details related to Fig 6 can be found in Appendix V of S1 Appendix.

#### 3.1.3 Summary

In sum, Korean speakers tend to increase the f0 values of on-focus syllables but decrease the f0 values of pre-focus and post-focus syllables for both monosyllables and disyllables. However, the changes did not reach statistical significance. For Japanese monosyllables, a significant increase of f0 in on-focus condition was observed, regardless of the stop types. In pre-focus and post-focus conditions, a significant decrease of f0 was found for both stop types. For Japanese HL disyllables, there was a significant increase of f0 in the on-focus condition and a decrease in the post-focus condition for both stop types. Only on voiced stop was there a significant decrease of f0 in the pre-focus condition. For Japanese LH disyllables, only a marginally significant increase of f0 was found in contrastive on-focus condition for both stop types, but not for other focus conditions.

### 3.2 Comparisons across stop types

#### 3.2.1 Korean

**Fig 7:**
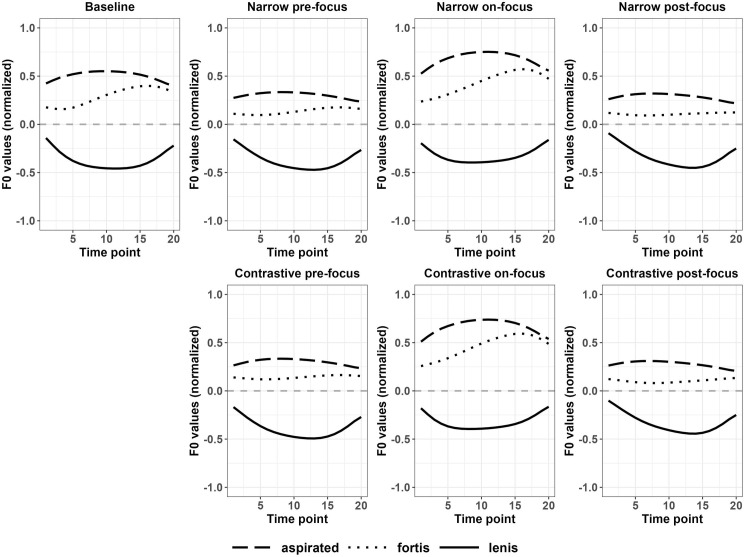
Normalized f0 contours of monosyllabic targets in various discourse conditions (Korean).

We have also conducted a comparison among various types of stops within each focus condition. For Korean monosyllabic targets, significant differences were found mostly in lenis vs. aspirated and lenis vs. fortis stop pairs across focus conditions. These results indicate that Korean speakers magnified consonant perturbation effects for these two syllable pairs. Fig 7 shows the f0 contours across focus conditions and Fig 9 (the upper panels) shows the significance regions. From Fig 7, the differences between f0 values after an aspirated and a fortis stop were quite small across focus conditions and did not reach significance. However, the differences between f0 values after an aspirated and a lenis stop became larger when the targets were in narrow on-focus conditions and so were the differences between f0 values after a fortis and a lenis stop. Moreover, in pre-focus and post-focus conditions, the regions of differences become marginally significant for lenis vs. aspirated syllable pairs, comparing to the baseline. The mean f0 values shown in Fig 7 can be found in Appendix IVA of S1 Appendix. These results suggested perturbation effects were magnified for on-focus words to contrast with pre-focus and post-focus words, which may serve as a way to signal focus marking.

**Fig 8:**
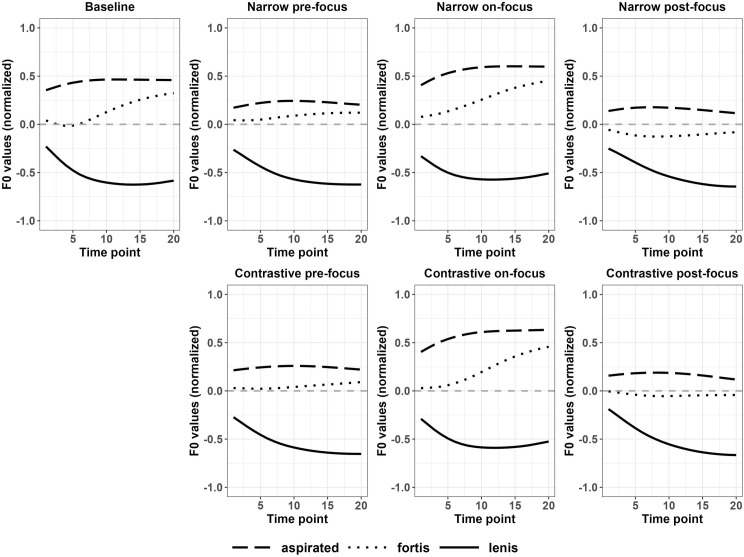
Normalized f0 contours of disyllabic targets under different discourse conditions (Korean).

**Fig 9:**
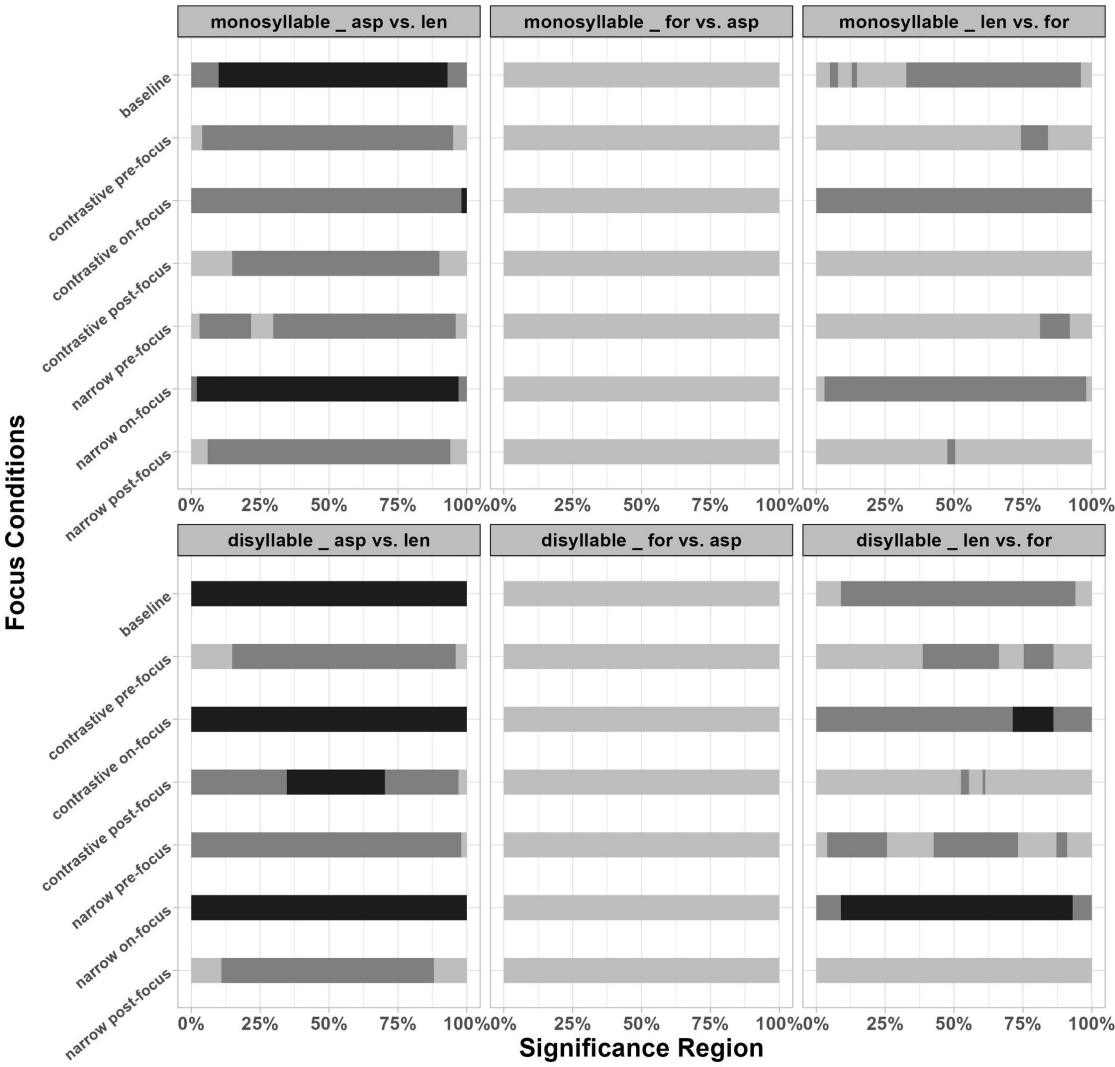
Significant regions between f0 after different stop types across focus conditions in Korean (the darkest part of the bar indicates regions of significant differences; the dark grey part indicates regions of marginal significance; the light grey part signals regions of no significant differences; asp = aspirated stop, len = lenis stop, for = fortis stop) (see Appendix VB in S1 Appendix for the statistics of significant region).

Korean disyllabic targets showed significant changes of f0 curves among stop types across focus conditions. As shown in Figs 8 and 9 (the lower panels), Korean speakers tended to magnify the differences between f0 contours after lenis vs. fortis stops to signal the focus while leaving the distance between fortis and aspirated stops relatively unchanged. For fortis vs. lenis stop pairs, the regions of significant differences were expanded in the on-focus conditions, while the differences between these curves shrunk or became non-significant in pre-focus and post-focus conditions, compared to the baseline. The differences between f0 values after lenis and aspirated stops remained significant for the entire f0 contours in on-focus conditions, while these differences were reduced in pre-focus and post-focus conditions. The mean f0 values shown in Fig 8 can be found in the Appendix IV of S1 Appendix. The regions of significant differences can be found in Appendix VB of S1 Appendix.

#### 3.2.2 Japanese

**Fig 10:**
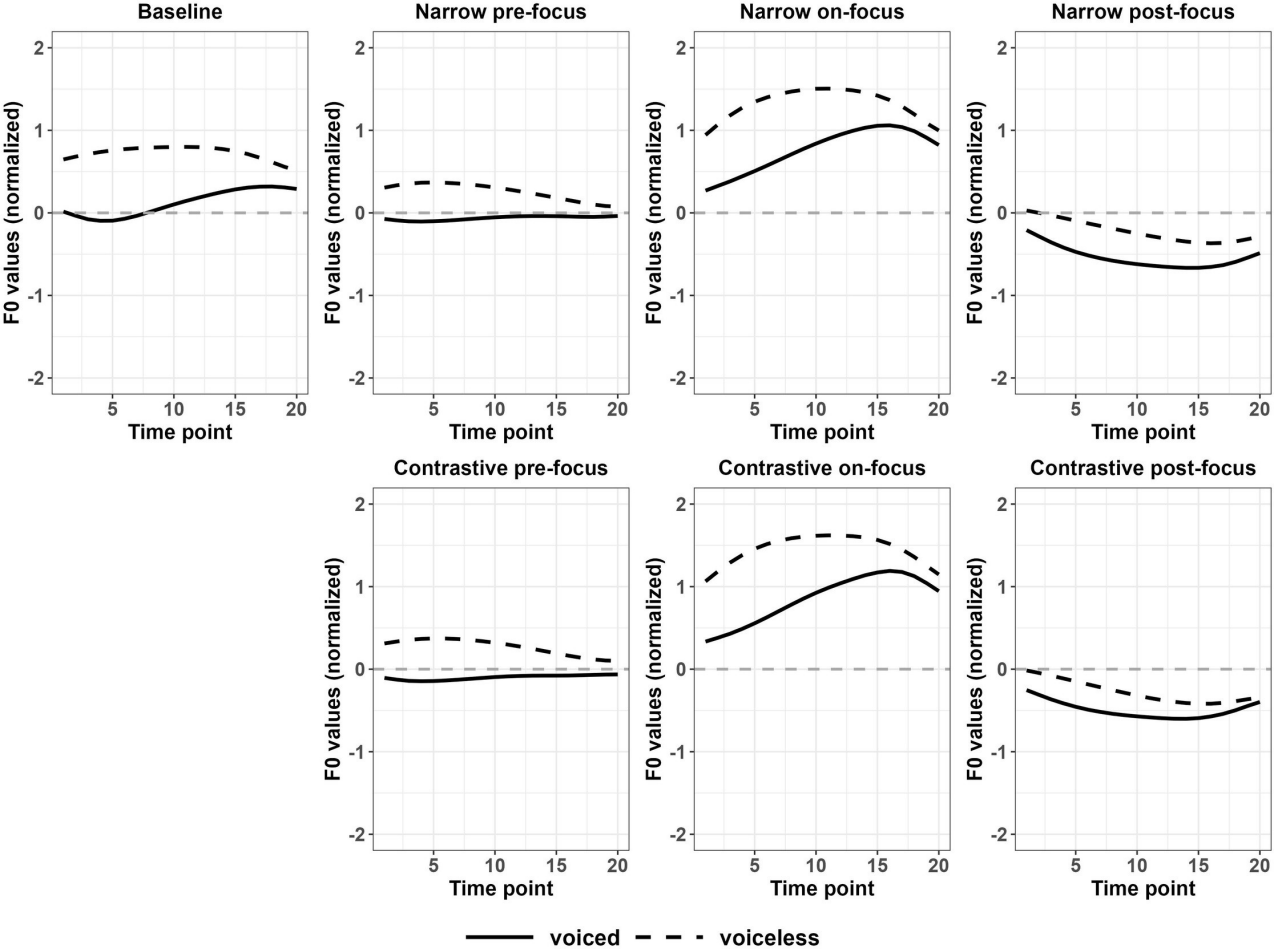
Normalized f0 contours of monosyllabic targets in various discourse conditions (Japanese).

For Japanese monosyllabic targets, as shown in Table 4, the f0 contours after voiced and voiceless stops were significantly different for narrow and contrastive on-focus conditions, compared to the baseline. Fig 10 shows that the f0 values after voiced and voiceless stops increased and the gap between the two f0 curves were also enlarged in on-focus conditions. The mean f0 values shown in Fig 10 can be found in Appendix IVB of S1 Appendix. In contrast, the gap between the two f0 curves after voiced and voiceless stops decreased for pre- and post-focus conditions.

**Table 4:** Significantly different regions between voiceless and voiced stops in various discourse conditions for (Japanese monosyllables).

	Baseline	Narrow pre-focus	Narrow on-focus	Narrow post-focus	Contrastive pre-focus	Contrastive on-focus	Contrastive post-focus
Voiced vs. voiceless stops	0~95.05%	0~85.15%	0~87.19%	0~100%	0~84.16%	0~100%	0~75.25%

**Fig 11:**
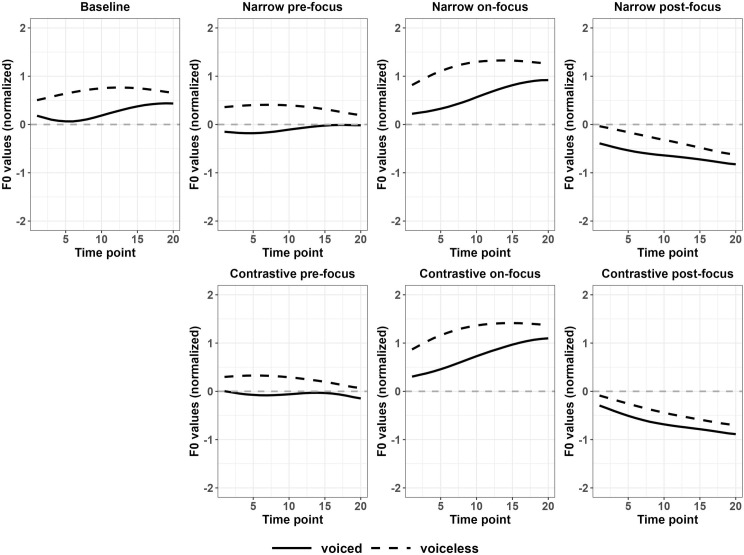
Normalized f0 contours of disyllabic HL targets between voiceless and voiced stops (Japanese).

**Fig 12:**
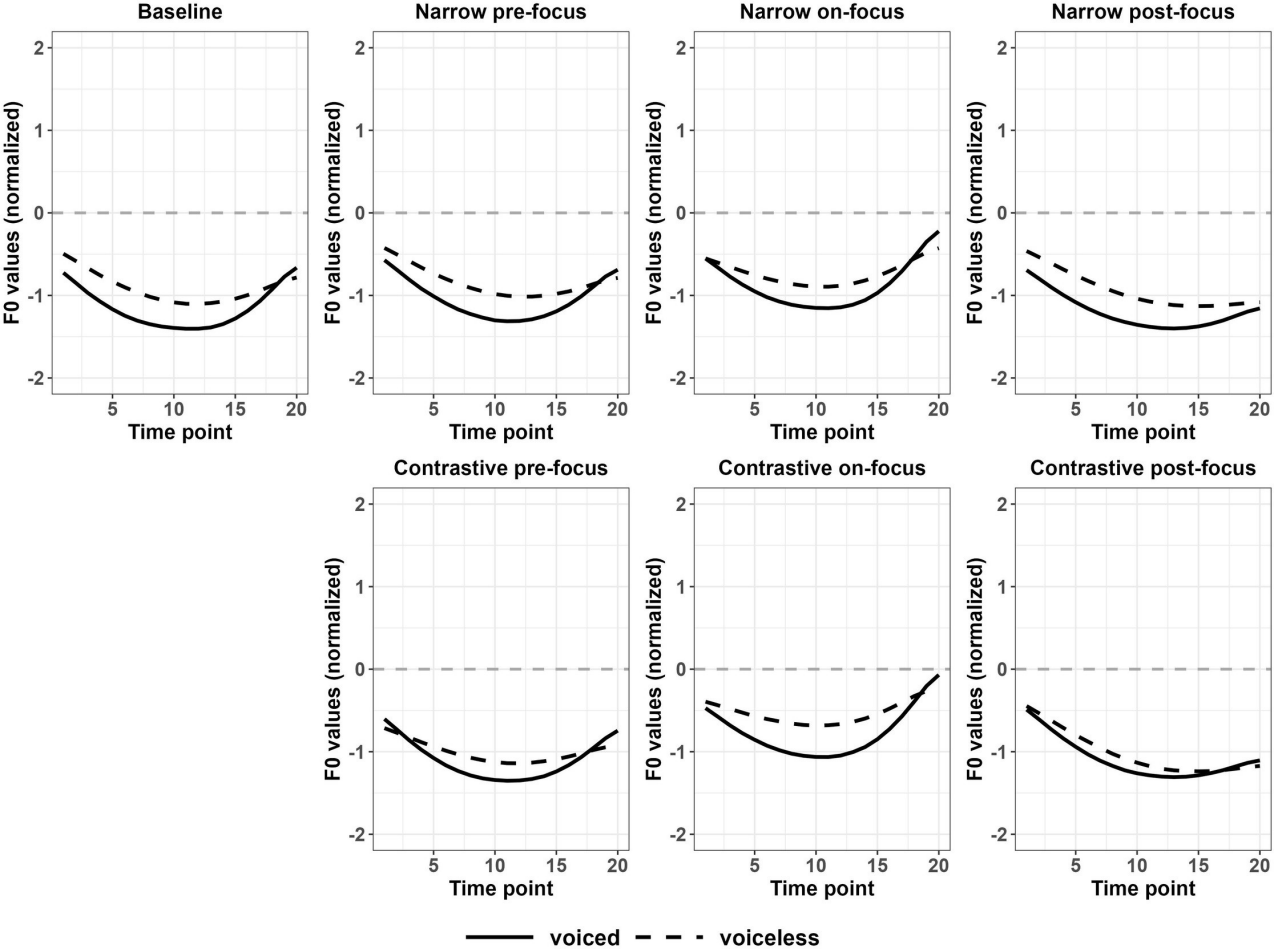
Normalized f0 contours of disyllabic LH targets between voiceless and voiced stops (Japanese).

The findings for differences between f0 curves after voiced and voiceless stops of Japanese disyllabic targets differ based on the pitch accent patterns. Figs 11 and 12 showed that the f0 contours after voiced and voiceless stops were enlarged for narrow on-focus conditions. Based on Table 5, for HL disyllables, the contrast of f0 between voiced and voiceless stops were significant for the entire f0 contours in all the focus conditions except for narrow post-focus condition, while the differences were not significant in the baseline condition. For LH disyllables, the contrast was insignificant in both the baseline and all other focused conditions. In the low pitch context, the perturbation effect was not manifested significantly compared to the high pitch context. The mean f0 values shown in Figs 11 and 12 can be found in Appendix IVD of S1 Appendix.

**Table 5:** Significantly different regions between voiceless and voiced stops in various discourse conditions (Japanese disyllables).

	Baseline	Narrow pre-focus	Narrow on-focus	Narrow post-focus	Contrastive pre-focus	Contrastive on-focus	Contrastive post-focus
HL	not sig.	0∼100%	0∼100%	not sig.	0∼100%	0∼100%	0∼100%
LH	not sig.	not sig.	not sig.	not sig.	not sig.	not sig.	not sig.

#### 3.2.3 Summary

In sum, regions of differences in f0 were magnified between aspirated and lenis stops, lenis and fortis stops to signal focus in Korean, while the difference in f0 between fortis and aspirated stops remained relatively stable for both monosyllabic and disyllabic targets. On the contrary, the f0 differences were significant after voiced and voiceless monosyllables in on-focus conditions of Japanese, but the differences were reduced in pre-focus and post-focus conditions. For HL disyllables, the f0 differences after voiced and voiceless stops were significantly enlarged compared to the f0 differences on the LH syllables.

### 3.3. F0 perturbation magnitude

#### 3.3.1 Korean

A linear mixed effects model was fitted with *FBDiff* (the mean f0 differences between the baseline and the focused condition) values as the response variable, *focus condition* and *stop type* as fixed effects, *subject* and *item* as random effects. Likelihood ratio tests were used to test the significance of the fixed effects and their interaction terms by adding each independent variable one at a time for comparisons until an optimal model is obtained.

For monosyllables, the optimal model was the one with *focus condition* (χ^2^ = 515.28; df = 5; *p* < 0.001), *stop type* (χ ^2^ = 20.50; df = 2; *p* < 0.001) and their interaction (χ^2^ = 257.96; df = 10; *p* < 0.001) as fixed effects. We carried out post-hoc tests using the “emmeans package” [67], to further interpret the interaction. Post-hoc analyses showed that *FBDiff* was not significantly different between aspirated and fortis stops, regardless of focus conditions. However, *FBDiff* of aspirated stop vs. lenis stop and fortis stop vs. lenis stop were significantly different in all focus conditions except for fortis stop vs. lenis stop in narrow focus condition (*p* = 0.13) (see Appendix VIA of S1 Appendix for the statistics of post-hoc analysis). As shown in Table 6, f0 values increased after aspirated and fortis monosyllables with a similar magnitude while f0 values after lenis stops showed changes of a smaller magnitude. Post-hoc analyses also showed that for an aspirated stop and a fortis stop, their *FBDiffs* of contrastive/narrow on-focus condition were significantly different from that of contrastive/narrow pre-focus or post-focus conditions while a lenis stop did not show significant differences in *FBDiff* between on-focus and pre-focus/post-focus conditions.

**Table 6:** Mean f0 perturbation magnitude between baseline and various focus conditions (FBDiff = focus–baseline) of Korean. The positive sign indicates an increase in f0, compared to the baseline and the negative sign indicates a decrease in f0.

FBDiff (Hz)	Contrastive on-focus	Contrastive pre-focus	Contrastive post-focus	Narrow on-focus	Narrow pre-focus	Narrow post-focus
**Aspirated monosyllable**	15.67	-36.42	-1.92	39.80	-36.97	-1.61
**Fortis monosyllable**	13.79	-29.25	-3.38	30.05	-27.31	-2.93
**Lenis monosyllable**	2.12	-4.96	4.47	0.18	-3.30	2.28
**Aspirated disyllables**	14	-31.5	-6.76	36.96	-31.33	-6.03
**Fortis disyllables**	7.88	-14.67	-7.76	25.59	-14.97	-15.07
**Lenis disyllables**	1.12	-1.52	2.06	-0.67	-0.02	1.29

The results of disyllables were similar to that of monosyllables, where *focus condition* (χ^2^ = 189.01; df = 5; *p* < 0.001), *stop type* (χ^2^ = 16.78; df = 2; *p* < 0.001) and their interaction (χ^2^ = 144.87; df = 10; *p* < 0.001) showed significance. The only difference is that *FBDiff* of a fortis stop and a lenis stop reached significance in post-focus condition, rather than on-focus and pre-focus conditions (see Appendix VIB in S1 Appendix for the statistics of post-hoc analyses).

#### 3.3.2 Japanese

A linear mixed effects model was fitted with *FBDiff* values as the response variable, *focus condition* and *stop type* as fixed effects, *subject* and *item* as random effects. The procedure of model fitting is the same as the model used in Korean data analysis.

For Japanese monosyllables, *focus condition* (χ^2^ = 1022.1; df = 5; *p* < 0.001), *stop type* (χ^2^ = 14.18; df = 1; *p* < 0.001) and their interaction (χ^2^ = 28.59; df = 5; *p* < 0.001) were significant. Post-hoc analyses showed that *FBDiffs* were significantly different between voiced and voiceless stops in contrastive pre-focus (*p =* 0.0046), contrastive post-focus (*p* < 0.001), narrow pre-focus (*p =* 0.01), and narrow post-focus (*p =* 0.015) conditions (see Appendix VIC in S1 Appendix). However, in two on-focus conditions, their differences were not significant, indicating similar magnitude of perturbation in f0 values after voiced and voiceless stops in on-focus conditions. Moreover, post-hoc analyses showed that, for both voiced and voiceless stops, the *FBDiffs* of contrastive/narrow on-focus conditions were significantly different from that of contrastive/narrow pre-focus or post-focus condition. From Table 7, it can be found that Japanese speakers tended to increase mean f0 in on-focus conditions and compress f0 in pre-focus and post-focus conditions.

**Table 7:** Mean f0 perturbation magnitude between baseline and different focus conditions (FBDiff = focus–baseline) of Japanese. Positive sign indicates an increase of f0, compared to the baseline. Negative sign indicates a decrease.

FBDiff (Hz)	Contrastive on-focus	Contrastive pre-focus	Contrastive post-focus	Narrow on-focus	Narrow pre-focus	Narrow post-focus
Voiced monosyllables	23.03	-20.24	-7.53	21.13	-21.30	-5.73
Voiceless monosyllables	24.56	-34.46	-17.14	19.63	-31.93	-16.65
Voiced LH disyllables	8.91	0.42	0.88	6.11	-1.75	2.27
Voiceless LH disyllables	7.98	-3.77	-2.69	3.99	-1.32	1.71
Voiced HL disyllables	14.37	-30.01	-11.78	9.63	-29.14	-11.31
Voiceless HL disyllables	19.53	-38.19	-17.51	16.70	-35.47	-12.47

For Japanese disyllables, we fitted the model with *focus condition*, *stop type*, *pitch accent* and their interactions as fixed effect. *Focus condition* (χ^2^ = 305.7; df = 5; *p* < 0.001), *pitch accent* (χ^2^ = 26.39; df = 1; *p* < 0.001) and their interactions (χ^2^ = 195.72; df = 5; *p* < 0.001) were significant, indicating that *FBDiff* of on-focus condition were significantly different from that of pre-focus and post-focus conditions. Post-hoc analyses showed that *FBDiff* was significantly different between LH and HL in contrastive/narrow pre-focus and contrastive/narrow post-focus conditions. However, *FBDiff* was not significantly different between LH and HL in contrastive/narrow on-focus conditions (see Appendix VID in S1 Appendix).

#### 3.3.3 Summary

In sum, for both Korean monosyllables and disyllables, f0 values increased in on-focus conditions with a larger magnitude after aspirated and fortis stops, compared to lenis stops. For Japanese monosyllables, f0 values increased after voiced and voiceless stops with a similar degree in on-focus conditions. For Japanese disyllables, f0 values increased with a bigger amplitude in disyllables with a pitch of HL than LH in on-focus conditions.

## 4. Discussion

Recall that the current study aims to answer three research questions. The first question is whether perturbation effects can be magnified to mark constituents under focus in Korean and Japanese. The second question examines whether perturbation effects were also compressed on constituents before and after the focus. The third question examines if f0 perturbation effects are realized differently in Korean and Japanese. The following sections attempt to answer all these three research questions.

### 4.1 The role of f0 perturbation in marking on-focus words

Recall that focus marking belongs to one case of linguistic prominence, where new information is highlighted [1]. Acoustic cues within a syllable may also be used for focus marking as a recent study showed lengthening of VOT and f0 perturbation in Korean were involved in focus marking [21]. However, this aspect is relatively less investigated on both monosyllables, disyllables in both narrow and contrastive focus with more advanced statistical methods. In general, our results on both Korean and Japanese further support that f0 perturbation is also involved in marking words under focus.

Specifically, we further analyzed how f0 contours were realized differently across focus conditions and across stop types using functional data analysis for the first time. Our results showed that the f0 contours between lenis vs. aspirated stops reached significance for almost the entire contour in the baseline condition, confirming the results from previous studies [17, 22]. In addition, our results in Korean using both monosyllables and disyllables showed similar results as reported by [21]. For Korean, no significant difference was found across focus conditions in both monosyllables and disyllables. However, when compared across stop types, Korean speakers were found to significantly enlarge the differences in f0 between fortis and lenis stops as well as aspirated and lenis stops in on-focus words from the initial part of the contour to the end reflected in the significant regions and the magnitude, indicating that they employed f0 perturbation as a cue to mark focus for both narrow and contrastive focus. It might be due to the fact that the VOT of the fortis stops is significantly shorter than the other two stop types [68] and thus f0 may not be used as a cue to differentiate the two stop types both in the baseline conditions and for on-focus conditions. In on-focus conditions, the VOT of a fortis stop is reported to be shortened (though only for 3ms) while that of an aspirated stop was lengthened more than a lenis stop, leading to an enhanced contrast of the two stops [21], which may render the changes in f0 contours less necessary.

On the contrary, Japanese speakers showed more changes in f0 contours across focus conditions. Nearly all the voiceless and voiced monosyllables in on-focus conditions (both narrow and contrastive) were significantly different from the baseline for the entire f0 contour. When compared across stop types, f0 contours after voiced and voiceless stops were significantly different from the initial part of f0 contour to almost the end of the contour in the on-focus condition. However, f0 contours were also significantly different in the baseline conditions, so we calculated the mean values to check the gap between f0 values after voiced and voiceless stops. The results showed that the gap between f0 values were enlarged for the first half of the contour in the on-focus condition. Hence, from the Japanese monosyllabic data, it is likely that Japanese speakers adopted a f0 raising strategy and f0 values increased after voiced and voiceless stops with a similar degree, compatible with previous Japanese studies (e.g., [15, 50]), where f0 values are reported to rise in on-focus words.

For Japanese disyllable data, pitch accent type played a role in f0 perturbation. In disyllables with HL pitch accent, f0 contours showed significant changes from the baseline condition, but LH disyllabic targets showed f0 changes to a lesser degree. Similar observation was also made when compared across stop types. For HL disyllables, the gap between f0 values after voiced and voiceless stops were enlarged for the entire f0 contour, but for the LH syllables, the gap remained similar. The finding that focus triggered a larger degree of perturbation effect in high than in low pitch context might suggest that when the pitch context is high and congruent with the f0 raising in on-focus position, the perturbation effect will be strengthened. Our findings also support the claim that pitch accent does not necessarily limit the perturbation effect [19] and perturbation effects can be used to indicate focus.

The Articulatory Phonology framework has been used to explain the observed VOT lengthening and perturbation effect in [21]. Their results support the argument that a focus gesture leads to the slowing of gestural activations and increment in gestural magnitude. Specifically, for the fortis and aspirated stops, a gesture HtenseC is modulated by the focus gesture so that f0 values become higher after the fortis and the aspirated stops. HtenseC gesture is one gesture that contributes to the f0 values of the vowel gesture during the gesture of tense consonants. Our results in Korean also showed that f0 values became more different between the lenis and aspirated as well as fortis and lenis stops in on-focus conditions, which might be due to the modulation so that the gap of f0 values in these pairs became larger. Moreover, Japanese also showed higher f0 values after voiced and voiceless stops in monosyllables and disyllables with the HL accent under focus. Since f0 values after voiced stops are usually much lower than those after voiceless stops, the increasing f0 values after the voiceless stops support that the focus gesture leads to an increment in gestural magnitude under the articulatory phonology framework [21]. Specifically, a gesture of HvoicelessC can be proposed, and this gesture will be modulated under the focus gesture, leading to an increase of f0 values. Moreover, the pitch accent may also play a role in that in the L tone context, the perturbation effects were not further modulated under focus.

In sum, our results provide new insights that sub-syllabic level acoustic cues such as f0 perturbation can also be employed in encoding communicative functions such as focus. The changes in f0 perturbation were similar for both narrow and contrastive focus in both languages. The encoding of the focal function may be affected by the encoding of other functions. For example, [50] stated that pitch accent plays a role in focus marking in Japanese, where f0 showed more changes in accented words than unaccented words. In addition, our results showed that the pitch accent can also affect the realization of f0 perturbation in a sub-syllabic level. It might also be due to different degrees of focus realization as reported by [50] or the effect of pitch accent on perturbation [49]. It is reported that in a neutral condition, a more salient effect of f0 perturbation in H context than in L context in word initial position. It is likely that in focus conditions, perturbation effects are also more salient in the H context due to the effect of pitch accent on perturbation.

### 4.2. F0 perturbation in pre- and post-focus positions

It has been proposed that focus marking may be related to a tri-zone [69], in which exaggerated acoustic cues such as f0 is witnessed in on-focus items while post-focus regions show evidence of compression in many languages such as Japanese and Korean, also examined in this study though post-focus compression is not a universal phenomenon [33]. Pre-focus items are argued to remain unchanged or show some compression. However, how sub-syllabic level f0 perturbation is realized in pre- and post-focus positions is relatively less examined.

Our results showed that f0 perturbation was often compressed in pre- and post-focus positions. Korean data showed fewer f0 contour differences after three types of stops in the pre- and post-focus positions compared to the on-focus positions, indicating a suppressing effect of f0 perturbation in those positions. Post-focus positions showed even more compression than pre-focus positions, indicating that compression of f0 perturbation in post-focus positions may be a more reliable strategy in focus marking compared to pre-focus compression.

Similar strategies were also found in our Japanese data. The differences between f0 contours in monosyllables after voiced and voiceless stops tended to decrease in pre- and post-focus positions. F0 perturbation tends to decrease in post-focus positions for Japanese disyllables bearing the HL accent. Previous studies [15, 70] showed that only accented syllables presented post-focus compression and [70] argued that when accented words exist within the focus domain, the realization of PFC is strengthened. Our results thus showed that f0 perturbation was also suppressed in post-focus positions following pitch accented syllables.

In sum, the sub-syllabic level f0 perturbation also showed some compression in both pre-focus and post-focus positions for both types of narrow and contrastive focus based on our data from Korean and Japanese. These results further suggest that f0 perturbation can play an important role in various types of focus marking.

### 4.3. Cross-linguistic f0 perturbation effects in focus marking

Since f0 can be employed to encode multiple communicative functions, it is likely that cross-linguistic differences of f0 perturbation in focus marking can be found when f0 is used to encode multiple functions. For example, tonal languages tend to suppress f0 changes for focus marking probably because the original f0 contours need to be maintained for lexical contrasting to some extent. Also, a tonal language may show f0 perturbation to a lesser degree, which may be due to the same need to contrast lexical meanings [55].

Korean is a language with a three-way contrast in stops and it has been reported that f0 started to play a more important role of distinguishing stops than VOT and it is proposed that Korean may be at a stage of quasi-tonogenesis [17, 22]. Specifically, [17] argues that VOT between lax and aspirated stops become similar, though there is no VOT change in the tense stops. The differences between lax and aspirated/tense stops are reflected in f0, where the mean f0 values after lax stops is significantly lower. Perception results also show that Seoul Korean listeners also rely on f0 more than VOT to distinguish lenis and aspirated stops [39]. For Korean, no significant difference was found across focus conditions in both monosyllables and disyllables, and yet Korean speakers significantly enlarge the differences in f0 between fortis and lenis stops as well as aspirated and lenis stops in on-focus words.

Compared to Korean, Japanese is less restricted in using f0 perturbation to indicate on-focus words in focus marking. Our results confirmed that unlike tonal languages showing a suppressing effect in f0 perturbation due to the necessity to maintain the f0 contour to indicate lexical contrasts, Japanese showed significant f0 perturbation effects despite the use of f0 to mark pitch accent in neutral focus conditions [19, 55]. As shown in the Korean and Japanese data, different languages may show various degrees of perturbation effects. It is reported that phonetic perturbation in a tonal language Chongming Chinese is to a lesser degree compared to that of Japanese [55]. For Korean, a language at a stage of quasi-tonogenesis, the perturbation effects are suppressed compared to Japanese. Despite the need to use f0 to mark the accent in Japanese, perturbation effects were less restricted in both the baseline and focus conditions. The perturbation effect is also magnified in on-focus targets in monosyllables and disyllables with the HL accent in Japanese.

The results from our study showed that whether perturbation effects are significant might not be necessarily related to the multiple functions of f0. Although f0 perturbation effects are more restricted in some tonal languages, which may be due to the multiple function of f0 in marking focus and lexical contrasts, our results in Japanese showed that the f0 perturbation effects were less restricted. However, Korean used f0 to differentiate stop types and showed a more restricted f0 perturbation effects. Moreover, both languages enlarged perturbation effects in on-focus positions and suppressed the effects in pre- or post-focus positions. Korean showed more significant changes across types compared to Japanese, which may be due to the fact that perturbation is suppressed more in the baseline condition. Future studies on other languages are called for to further understand perturbation effects in pre-, on- and post-focus conditions and especially the relationship between f0 perturbation and focus in languages with and without post-focus compression.

## 5. Conclusions

The present study provides evidence from Tokyo Japanese and Seoul Korean that sub-syllabic level f0 perturbation effects may also be used in focus marking as f0 perturbation effects were magnified in on-focus positions and compressed in pre- and post-focus positions for both narrow and contrastive focus. Our results also showed some differences in the degree of perturbation effects in the two languages examined. Seoul Korean tend to show perturbation effects to a lesser degree compared to a pitch-accent language Tokyo Japanese. In addition, our results showed that pitch accent affected the realization of f0 perturbation in various focus conditions.

## Supporting information

S1 File Korean raw data. Raw Korean f0 data extracted from each speaker.
(CSV)

S2 File Korean log data. Log-normalization of the raw data.
(CSV)

S3 File Korean trimmed monosyllables. Trimmed log-normalization data of Korean monosyllables.
(CSV)

S4 File Korean trimmed first syllables of disyllables. Trimmed log-normalization data of the first syllables in Korean disyllables.
(CSV)

S5 File Korean trimmed disyllables. Trimmed log-normalization data of both syllables in Korean disyllables.
(CSV)

S6 File Japanese data. Japanese raw f0 data and log-normalized data for each speaker.
(CSV)

S7 File Japanese trimmed monosyllables. Trimmed log-normalization data of Japanese monosyllables.
(CSV)

S8 File Japanese monosyllable mean. Mean log-normalization f0 value per syllable of Japanese monosyllables.
(CSV)

S9 File Japanese trimmed disyllables. Trimmed log-normalization data of Japanese disyllables.
(CSV)

S10 File Japanese disyllable mean. Mean log-normalization f0 value per syllable of Japanese disyllables.
(CSV)

S1 Appendix (DOCX)
