# Supplementary material for: The f0 perturbation effects in focus marking: Evidence from Korean and Japanese
Source: PLoS One. 2023 Mar 23;18(3):e0283139. doi: 10.1371/journal.pone.0283139 (PMC10035888; doi:10.1371/journal.pone.0283139)
Supplement: S1 Appendix — (DOCX) [file pone.0283139.s011.docx]

**Appendix**

Appendix I. Stimuli

IA. Korean Stimuli

IA1. Monosyllabic word list

| **Consonant/Vowel** | **/a/** | **/e/** | **/o/** |
| --- | --- | --- | --- |
| **/p/** | 바 | 베 | 보 |
| **/p͈/** | 빠 | 뻬 | 뽀 |
| **/pʰ/** | 파 | 페 | 포 |
| **/t/** | 다 | 데 | 도 |
| **/t͈/** | 따 | 떼 | 또 |
| **/tʰ/** | 타 | 테 | 토 |
| **/k/** | 가 | 게 | 고 |
| **/k͈/** | 까 | 께 | 꼬 |
| **/kʰ/** | 카 | 케 | 코 |

IA2. Disyllabic word list

| Word | IPA |
| --- | --- |
| 가루 | /kalu/ |
| 까락 | /k͈alak/ |
| 카레 | /kʰale/ |
| 바리 | /pali/ |
| 빠네 | /p͈ane/ |
| 파리 | /pʰali/ |
| 다리 | /tali/ |
| 따리 | /t͈ali/ |
| 타르 | /tʰalɯ/ |

IB. Japanese Stimuli

IB1. Monosyllabic word list

| **Consonant/Vowel** | **/a/** | **/e/** | **/o/** |
| --- | --- | --- | --- |
| **/p/** | ぱ | ぺ | ぽ |
| **/t/** | た | て | と |
| **/k/** | か | け | こ |
| **/b/** | ば | べ | ぼ |
| **/d/** | だ | で | ど |
| **/ɡ/** | が | げ | ご |

IB2. Disyllabic word list (Because it is very hard for the participants to produce the desired pitch accent using non-sense disyllabic words, we chose real words bearing the desired accent patterns to elicit natural pitch accent.)

| Pitch accent: LH | | Pitch accent: HL | |
| --- | --- | --- | --- |
| word | **IPA** | **Word** | **IPA** |
| 滝 “waterfall” | /taki/ | 凧 “kite” | /tako/ |
| 抱く “embrace” | /dakɯ/ | 舵機 “steering gear” | /daki/ |
| 敵 “enemy” | /teki/ | 梃 “lever” | /teko/ |
| 出来 “craftsmanship” | /deki/ | 凸 “convex” | /deko/ |
| 徳 “virtue” | /tokɯ/ | 朱鷺 “Japanese crested ibis” | /toki/ |
| 毒 “poison” | /dokɯ/ | 土器 “earthenware” | /doki/ |

Appendix II. Participant information

IIA. Overview of participant information

IIA1. Korean participants

Number of Korean participants, their average age of language acquisition (and standard deviations), and their average self-reported language scores (5 points each) (and standard deviations)

| Language | Num | AoA | Writing | Reading | Speaking | Listening |
| --- | --- | --- | --- | --- | --- | --- |
| Mandarin | 15 | 22.1 (5.9) | 0.9 (0.3) | 1.2 (0.6) | 1.3 (0.5) | 1.2 (0.4) |
| Cantonese | 4 | 23.3 (0.6) | 1 (0) | 1 (0) | 1 (0) | 1 (0) |
| English | 17 | 9.8 (5.0) | 4.1 (0.6) | 4.1 (0.6) | 4.2 (0.8) | 4.1 (0.9) |
| Japanese | 2 | 22.5 (9.2) | 3 (0) | 4 (0) | 4 (0) | 4 (0) |

IIA2. Japanese participants

Number of Japanese participants, their average age of language acquisition (and standard deviations), their average self-reported language scores (5 points each) (and standard deviations)

| Language | Num | AoA | Writing | Reading | Speaking | Listening |
| --- | --- | --- | --- | --- | --- | --- |
| Mandarin | 4 | 23.3 (5.0) | 1.5 (0.6) | 2 (1.4) | 1.5 (0.6) | 1.8 (1.0) |
| Cantonese | 2 | 33.5 (4.9) | 1 (0) | 1 (0) | 1 (0) | 1 (0) |
| English | 13 | 9.8 (4.0) | 3 (0.8) | 3.1 (0.8) | 3.2 (0.9) | 3.1 (0.8) |
| Korean | 1 | 20 | 4 | 4 | 4 | 4 |

IIB. Language background of stimuli recorders

IIB1. Language Background of the Korean Speaker for Stimuli Recording (5 points for each language score)

| Language | AoA | Writing score | Reading score | Speaking score | Listening score |
| --- | --- | --- | --- | --- | --- |
| Mandarin | 20 | 1 | 1 | 2 | 2 |
| Cantonese | 21 | 1 | 1 | 1 | 1 |
| English | 10 | 4 | 4 | 5 | 5 |
| Japanese | 13 | 2 | 1 | 3 | 4 |

IIB2. Language Background of the Japanese Speaker for Stimuli Recording (5 points for each language score)

| Language | AoA | Writing score | Reading score | Speaking score | Listening score |
| --- | --- | --- | --- | --- | --- |
| Mandarin | 25 | 2 | 2 | 2 | 2 |
| English | 15 | 3 | 3 | 3 | 3 |

Appendix III. Examples of Functional T-test Analysis

IIIA. Korean sample

1. Korean sample
   1. F0 contours after fortis stops in monosyllables: narrow on-focus vs. baseline

Observed t-values:

0.4396503 0.4566256 0.4733210 0.4892814 0.5041257 0.5175683 0.5294431 0.5396834 0.5482543 0.5551515 0.5604129 0.5641232 0.5664153 0.5674691 0.5674919 0.5666963 0.5652837 0.5634351 0.5613027 0.5590097 0.5566589 0.5543406 0.5521390 0.5501325 0.5483841 0.5469320 0.5457836 0.5449132 0.5442664 0.5437706 0.5433451 0.5429108 0.5423979 0.5417557 0.5409647 0.5400315 0.5389788 0.5378374 0.5366412 0.5354100 0.5341436 0.5328302 0.5314544 0.5300050 0.5284884 0.5269352 0.5253911 0.5239088 0.5225423 0.5213376 0.5203174 0.5194844 0.5188302 0.5183423 0.5180122 0.5178394 0.5178262 0.5179678 0.5182465 0.5186283 0.5190702 0.5195308 0.5199770 0.5203912 0.5207773 0.5211567 0.5215549 0.5219897 0.5224633 0.5229569 0.5234350 0.5238667 0.5242355 0.5245461 0.5248305 0.5251466 0.5255489 0.5260641 0.5266795 0.5273333 0.5279124 0.5282836 0.5283275 0.5279486 0.5270836 0.5257081 0.5238138 0.5213603 0.5182606 0.5143783 0.5095326 0.5035334 0.4962654 0.4877271 0.4780415 0.4674554 0.4563053 0.4448819 0.4333501 0.4217400 0.4099499

The maximum 0.008 critical value: 3.246988

Pointwise 0.008 critical values:

2.106167 2.063325 2.011121 1.960701 1.901856 1.854451 1.768560 1.684119 1.700462 1.757868 1.865657 1.876326 1.888871 1.803464 1.930610 1.922704 1.908970 1.918244 1.880380 1.894618 1.888824 1.892691 1.875068 1.783440 1.821160 1.756462 1.800886 1.803542 1.895663 1.900038 1.913968 1.858700 1.861007 1.856194 1.839566 1.812903 1.829885 1.821382 1.844173

1.878864 1.902704 1.935419 1.973416 1.972592 1.962435 2.059608 2.053634 2.051086 2.067342 2.055701 2.078939 2.087820 2.062376 2.115860 2.109943 2.086901 2.098295 2.116080 2.092000 2.054638 2.001531 1.962491 2.119526 2.125056 2.095738 2.057580 2.059466 2.044215 1.960535 1.951992 1.920518 1.943369 1.952203 2.039985 2.048592 2.001578 1.961686 1.891665

1.859669 1.873734 1.875893 1.893443 1.946855 1.950788 1.895862 1.924139 1.943924 1.930466 2.006547 1.977068 1.980967 2.024878 2.024228 2.068726 2.167980 2.172658 2.081490 2.172599 2.248286 2.254156 2.242447

- 1. F0 contours after fortis stop in disyllables: narrow on-focus vs. baseline

Observed t-values:

0.4632963 0.4790991 0.4944584 0.5090269 0.5224728 0.5344930 0.5448327 0.5532930 0.5597235 0.5640430 0.5662610 0.5664875 0.5649280 0.5618576 0.5575787 0.5523834 0.5465302 0.5402328 0.5336599 0.5269435 0.5201923 0.5135047 0.5069782 0.5007126 0.4948002 0.4893149 0.4843064 0.4797988 0.4757906 0.4722544 0.4691454 0.4664101 0.4639951 0.4618561 0.4599713 0.4583353 0.4569484 0.4558098 0.4549131 0.4542368 0.4537436 0.4533879 0.4531247 0.4529173 0.4527497 0.4526319 0.4525897 0.4526564 0.4528673 0.4532496 0.4538049 0.4545123 0.4553370 0.4562389 0.4571834 0.4581592 0.4591755 0.4602510 0.4614048 0.4626515 0.4639952 0.4654301 0.4669489 0.4685516 0.4702539 0.4720879 0.4740906 0.4762909 0.4786991 0.4812998 0.4840537 0.4869112 0.4898223 0.4927448 0.4956533 0.4985435 0.5014195 0.5042738 0.5070718 0.5097392 0.5121554 0.5141762 0.5156616 0.5164860 0.5165497 0.5157892 0.5141579 0.5115798 0.5079317 0.5030402 0.4966909 0.4886684 0.4788397 0.4672028 0.4539078 0.4392595 0.4236825 0.4076088 0.3913914 0.3752718 0.3593662

The maximum 0.008 critical value: 3.528315

Pointwise 0.008 critical values:

2.352461 2.332173 2.373169 2.417135 2.449768 2.522162 2.558662 2.644401 2.770248 2.880162 2.971066 3.040915 3.088922 3.115415 3.100495 3.052641 2.987514 2.907571 2.815142 2.712377 2.743816 2.725122 2.683514 2.635541 2.557074 2.432453 2.370553 2.306645 2.297982 2.279731 2.194811 2.096473 2.096551 2.146587 2.187570 2.218531 2.238693 2.227300 2.206831 2.168169 2.169810 2.176367 2.189490 2.174586 2.172361 2.262792 2.301206 2.325978 2.400744 2.451907 2.491996 2.520380 2.556820 2.628198 2.687285 2.733821 2.802620 2.872086 2.874282 2.831775

2.791970 2.746075 2.727582 2.709999 2.699282 2.675553 2.639822 2.593117 2.679546 2.704706 2.720918 2.727325 2.722983 2.694622 2.602071 2.498048 2.382849 2.439706 2.494453 2.523808 2.460798 2.551139 2.654374 2.740176 2.757550 2.716472 2.649516 2.556011 2.428392 2.411232 2.362088 2.318981 2.363870 2.405717 2.395237 2.342182 2.279362 2.232525 2.298581 2.446396 2.576832


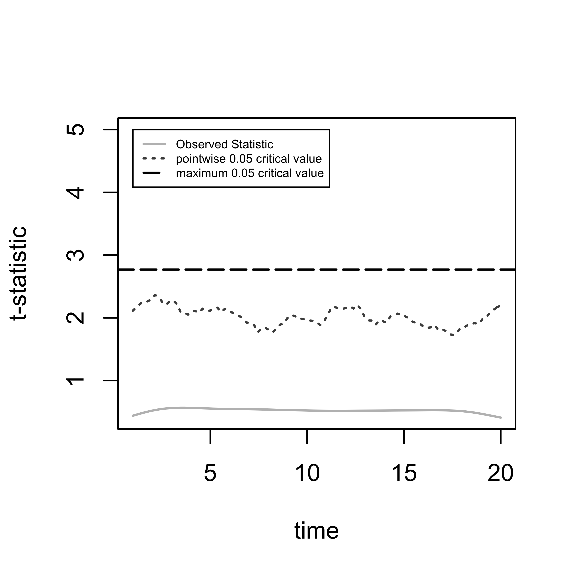

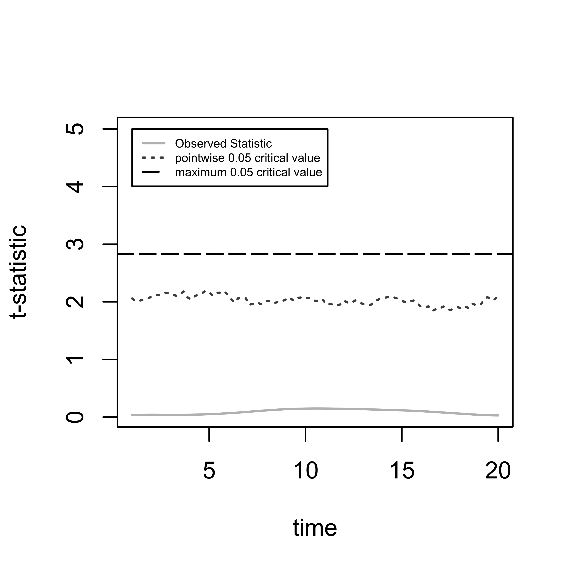


1. (B)

Fig A1. Functional data analysis of f0 contours after fortis stops produced by Korean speakers in contrastive on-focus and baseline condition (A): f0 contours after fortis stops in monosyllables; (B): f0 contours after fortis stops in disyllables; The solid line stands for the observed statistics. The dotted line stands for the pointwise 0.05 critical values and the dashed line stands for maximum 0.05 critical values.

IIIB. Japanese sample

a. Voiced stop in monosyllable: narrow on-focus vs. baseline

Observed t-values:

| 3.916018 4.201137 4.477535 4.744774 5.002206 5.249090 5.484741 5.708718 5.920926 6.121512 |
| --- |
| 6.310727 6.488820 6.655967 6.812301 6.957933 7.092955 7.217439 7.331464 7.435244 7.529214 7.614031 7.690576 7.759960 7.823382 7.881944 7.936637 7.988335 8.037809 8.085756 8.132865 |
| 8.179830 8.227344 8.276099 8.326750 8.379805 8.435579 8.494203 8.555625 8.619646 |
| 8.686049 8.754686 8.825460 8.898314 8.973212 9.050106 9.128911 9.209494 9.291689 9.375300 |
| 9.460067 9.545555 9.6311039.715790 9.798398 9.877424 9.951235 10.018159 10.076519 10.124671 10.161076 10.184376 10.193451 10.187442 10.165777 10.128168 10.074589 10.005254 9.920644 |
| 9.821542 9.709054 9.584641 9.450180 9.307929 9.160450 9.010546 8.861099 8.714537 8.572571 |
| 8.436201 8.305751 8.180946 8.061302 7.946463 7.836246 7.730654 7.629893 7.534287 7.444124 |
| 7.359644 7.281064 7.208582 7.142316 7.082083 7.027301 6.976941 6.929482 6.882930 6.835212 6.784427 6.728930 6.667438 |

The maximum 0.008 critical value: 3.548242

Pointwise 0.008 critical values:

2.972951 2.960668 2.953892 2.961509 2.963366 2.960674 2.954570 2.946092 2.985003 3.024021 3.059199 3.090847 3.119132 3.144216 3.161744 3.170472 3.177756 3.183879 3.189124 3.193740 3.197947 3.201939 3.205890 3.209947 3.214219 3.218781

3.223676 3.228918 3.234512 3.240488 3.246910 3.253870 3.261488 3.269892 3.279139 3.289187 3.299895 3.311028 3.322269

3.333347 3.344110 3.354538 3.364733 3.374918 3.385343 3.396174 3.407478 3.419226 3.431290 3.443475 3.455346 3.466386

3.477392 3.488240 3.498804 3.508943 3.518507 3.526666 3.533455 3.539062 3.543372 3.546265 3.547621 3.547315 3.545217

3.541207 3.535184 3.527078 3.516853 3.504508 3.490089 3.473683 3.455424 3.435498 3.414141 3.391636 3.368274 3.344318

3.319994 3.295485 3.270918 3.246392 3.222001 3.197841 3.174014 3.150635 3.127824 3.105699 3.084372 3.063955 3.044563

b. Voiced stop in HL disyllable: narrow on-focus vs. baseline

Observed t-values:

2.994137 3.015209 3.036471 3.057922 3.079562 3.101391 3.123408 3.145613 3.168004 3.190581 3.213342 3.236285 3.259408 3.282711 3.306190 3.329842 3.353666 3.377657 3.401813 3.426130 3.450604 3.475230 3.500005 3.524922 3.549977 3.575164 3.600477 3.625909 3.651455 3.677106 3.702855 3.728694 3.754616 3.780610 3.806668 3.832780 3.858935 3.885124 3.911334 3.937553 3.963769 3.989970 4.016140 4.042266 4.068333 4.094326 4.120227 4.146021 4.171690 4.197216 4.222580 4.247763 4.272746 4.297508 4.322028 4.346286 4.370259 4.393925 4.417263 4.440248 4.462858 4.485070 4.506861 4.528207 4.549084 4.569469 4.589339 4.608670 4.627440 4.645624

4.663201 4.680149 4.696445 4.712068 4.726998 4.741215 4.754698 4.767430 4.779393 4.790571 4.800946 4.810506 4.819237 4.827125 4.834160 4.840332 4.845633 4.850055 4.853592 4.856240 4.857996 4.858858 4.858827 4.857904 4.856091 4.853394 4.849819 4.845372 4.840063 4.833901 4.826899

The maximum 0.008 critical value: 2.90357

Pointwise 0.008 critical values:

2.803884 2.806902 2.809830 2.812665 2.815405 2.818045 2.820582 2.823012 2.825332 2.827538 2.829626 2.831592 2.833432 2.835143 2.836720

2.838158 2.839454 2.840604 2.841602 2.842444 2.843127 2.843644 2.843992 2.844167 2.844163 2.843976 2.843601 2.843033 2.842268 2.841302

2.840129 2.838745 2.837145 2.835325 2.833281 2.831007 2.828501 2.825756 2.822771 2.819540 2.816059 2.812325 2.808335 2.804084 2.799569

2.794786 2.789732 2.784405 2.778800 2.772915 2.766747 2.760293 2.753552 2.746521 2.739198 2.731582 2.723671 2.717668 2.719341 2.720769

2.721947 2.722871 2.718317 2.711884 2.705169 2.703290 2.710373 2.712967 2.712549 2.711847 2.710637 2.702827 2.705667 2.711841 2.717726

2.723316 2.728606 2.733591 2.738267 2.742629 2.746673 2.750396 2.753794 2.756863 2.759602 2.762008 2.764079 2.765813 2.767210 2.767897

2.768122 2.768009 2.767558 2.766771 2.765648 2.764191 2.762402 2.760283 2.757836 2.755065 2.751972

- 1. Voiceless stop in HL disyllable: narrow on-focus vs. baseline

Observed t-values:

2.327616 2.648744 2.737996 2.711570 2.710540 2.762343 2.825088 2.869848 2.908476 2.953701 2.993392 3.004642 2.993615 2.994000 3.024635 3.069437 3.086960 3.050750 2.977579 2.893425 2.803433 2.698640 2.584045 2.489361 2.442327 2.449212 2.491068 2.533444 2.556989 2.569079 2.580637 2.588920 2.586756 2.588375 2.620042 2.684890 2.748572 2.764941 2.738432 2.724888 2.760947 2.833764 2.889638 2.889470 2.867864 2.882700 2.952055 3.042765 3.096589 3.095002 3.071731 3.055193 3.040307 3.002550 2.936563 2.867671 2.818201 2.783965 2.737213 2.658374 2.565014 2.489879 2.452247 2.453431 2.488282 2.556951 2.653239 2.742672 2.766412 2.706418 2.631234 2.614124 2.671109 2.761282 2.817561 2.825231 2.838102 2.896976 2.987872 3.050376 3.044848 3.014081 3.019795 3.076707 3.142707 3.150960 3.084829 2.986170 2.893200 2.813503 2.736537 2.660652 2.601217 2.574050 2.584020 2.624985 2.686138 2.742952 2.722125 2.526298 2.173917

Pointwise values:

2.106442 2.142440 2.257588 2.083784 2.027907 2.092780 2.122250 2.190742 2.285087 2.343913 2.220639 2.166056 2.141680 2.117048 2.072460 1.988853 1.997584 1.871104 1.688179 1.760764 1.867360 1.855939 1.849439 2.008874 1.984936 1.954716 1.949046 2.128710 2.050855 1.996322 1.923361 1.978214 2.054417 2.009242 1.942171 2.004265 1.951010 1.814837 2.028828 2.033388 2.023186 1.874798 1.854594 2.068223 2.098946 1.963717 1.808192 1.727250 1.816195 1.819667 1.781277 1.700925 1.733278 1.860248 2.000999 1.973234 1.802701 1.839359 1.996864 2.029163 1.951845 1.975876 1.930589 2.056523 2.098551 2.124505 2.055640 1.935331 2.105614 2.011264 2.091388 2.125127 2.081729 2.027973 2.100274 2.078182 2.271026 2.372364 2.277897 2.091258 2.097124 2.146843 2.011655 2.091785 2.094401 1.894500 1.748839 1.756067 1.832241 1.890307 1.903327 1.914439 2.055101 2.020090 2.110918 1.910587 2.016252 2.041261 2.055403 1.894939 1.845335

The maximum 0.008 critical value: 4.052534

Pointwise 0.008 critical values:

2.594084 2.371672 2.333713 2.544720 2.476868 2.389548 2.294343 2.323108 2.613051 2.707942 2.591147 2.585338 2.716515 2.686934 2.815521

2.768512 2.909629 3.092242 3.244971 3.181628 3.091484 2.950510 2.765137 2.662957 2.621513 2.576834 2.560952 2.673125 2.635201 2.574830

2.588265 2.684343 2.576538 2.560924 2.600891 2.424450 2.340703 2.478638 2.592630 2.537351 2.594365 2.715574 2.779253 3.046113 2.986769

2.721969 2.682777 2.737324 2.637132 2.732093 2.716540 2.756283 2.730458 2.671083 2.551663 2.756515 2.833620 2.807287 2.672928 2.501921

2.542296 2.716048 2.817174 2.838814 2.708932 2.844595 2.900661 2.715548 2.571739 2.630075 2.460209 2.376270 2.509725 2.470284 2.361930

2.349076 2.403357 2.533671 2.598388 2.622044 2.624458 2.575945 2.675709 2.743337 2.835476 3.003653 3.253699 3.253878 3.217152 3.066998

2.843083 2.753725 2.676723 2.615129 2.670050 2.606154 2.706638 2.681292 2.489587 2.517312 2.585694


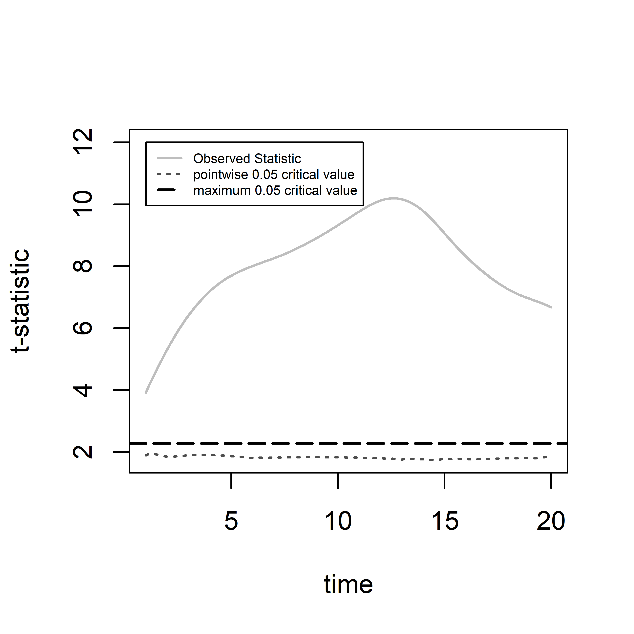

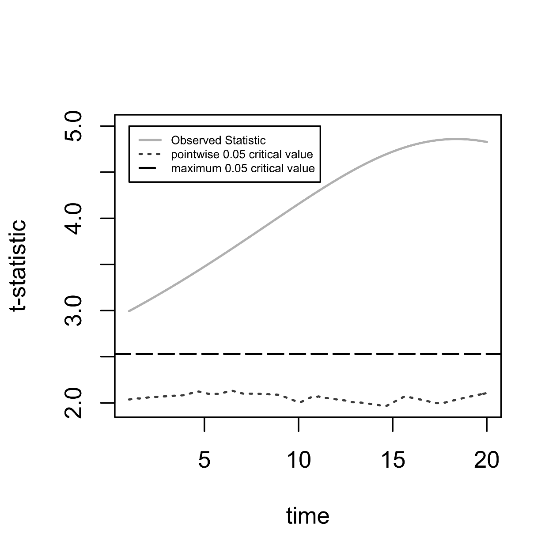


1. (B)


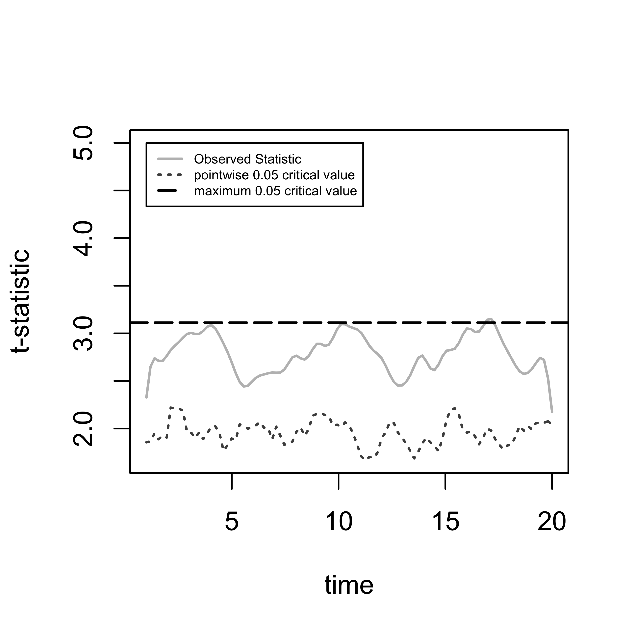


(C)

Fig A2. Functional data analysis of f0 contours after voiced and voiceless stops produced by Japanese speakers in narrow on-focus and baseline condition (A): voiced stop in monosyllables; (B): voiced stop in HL disyllables; (C) voiceless stop in HL disyllable; The solid line stands for the observed statistics. The dotted line stands for the pointwise 0.05 critical values and the dashed line stands for maximum 0.05 critical values.

Appendix IV. Mean normalized f0 values over 20 time points

IVA. Korean monosyllables

| **Stop** | **Focus** | **Normalized Time Points** | | | | | | | | | |
| --- | --- | --- | --- | --- | --- | --- | --- | --- | --- | --- | --- |
|  |  | **1/11** | **2/12** | **3/13** | **4/14** | **5/15** | **6/16** | **7/17** | **8/18** | **9/19** | **10/20** |
| **Aspirated** | **Baseline** | 0.423945 | 0.455239 | 0.482594 | 0.503425 | 0.519865 | 0.53257 | 0.542009 | 0.547986 | 0.55073 | 0.550908 |
|  |  | 0.549097 | 0.545241 | 0.538412 | 0.528636 | 0.515428 | 0.498041 | 0.475977 | 0.449317 | 0.420811 | 0.397292 |
|  | **Contrastive**  **pre-focus** | 0.264284 | 0.282671 | 0.299796 | 0.312892 | 0.322525 | 0.329108 | 0.332548 | 0.333782 | 0.332788 | 0.329877 |
|  |  | 0.325551 | 0.320106 | 0.313531 | 0.305181 | 0.295794 | 0.28535 | 0.272919 | 0.259654 | 0.245019 | 0.234361 |
|  | **Contrastive**  **on-focus** | 0.510086 | 0.563099 | 0.609094 | 0.644957 | 0.672103 | 0.694428 | 0.711479 | 0.724416 | 0.732762 | 0.737391 |
|  |  | 0.739011 | 0.737081 | 0.730769 | 0.720225 | 0.703963 | 0.681754 | 0.652026 | 0.615274 | 0.573307 | 0.53834 |
|  | **Contrastive**  **post-focus** | 0.262794 | 0.277098 | 0.290033 | 0.299442 | 0.305362 | 0.309058 | 0.3097 | 0.308936 | 0.306081 | 0.301651 |
|  |  | 0.296476 | 0.290299 | 0.283068 | 0.275228 | 0.266112 | 0.255802 | 0.243926 | 0.230506 | 0.216629 | 0.20689 |
|  | **Narrow**  **pre-focus** | 0.274316 | 0.289429 | 0.303546 | 0.314823 | 0.323116 | 0.329213 | 0.332843 | 0.334441 | 0.333829 | 0.331373 |
|  |  | 0.327849 | 0.322852 | 0.316063 | 0.307835 | 0.297732 | 0.286644 | 0.273838 | 0.259664 | 0.245449 | 0.235609 |
|  | **Narrow**  **on-focus** | 0.525411 | 0.576989 | 0.622729 | 0.658279 | 0.686075 | 0.708538 | 0.725776 | 0.738404 | 0.746005 | 0.750495 |
|  |  | 0.751929 | 0.749585 | 0.743546 | 0.733198 | 0.717184 | 0.695295 | 0.666385 | 0.630278 | 0.589662 | 0.556728 |
|  | **Narrow**  **post-focus** | 0.260828 | 0.277977 | 0.29357 | 0.305014 | 0.312657 | 0.317537 | 0.319608 | 0.319526 | 0.317703 | 0.314204 |
|  |  | 0.309609 | 0.303671 | 0.296945 | 0.288954 | 0.279555 | 0.268565 | 0.256112 | 0.24199 | 0.227734 | 0.218124 |
| **Lenis** | **Baseline** | -0.14159 | -0.21548 | -0.28373 | -0.33722 | -0.37877 | -0.408 | -0.42898 | -0.44218 | -0.45079 | -0.45601 |
|  |  | -0.45815 | -0.45773 | -0.45402 | -0.44495 | -0.42889 | -0.40367 | -0.36844 | -0.32331 | -0.26921 | -0.22054 |
|  | **Contrastive**  **pre-focus** | -0.16828 | -0.22168 | -0.27545 | -0.32292 | -0.36496 | -0.3997 | -0.42708 | -0.4482 | -0.46448 | -0.47735 |
|  |  | -0.48651 | -0.49217 | -0.49346 | -0.48804 | -0.47333 | -0.44892 | -0.41336 | -0.36753 | -0.31439 | -0.27098 |
|  | **Contrastive**  **on-focus** | -0.18024 | -0.2433 | -0.29918 | -0.33977 | -0.36808 | -0.38433 | -0.39253 | -0.39506 | -0.3946 | -0.39203 |
|  |  | -0.38751 | -0.38089 | -0.3719 | -0.36033 | -0.34413 | -0.32133 | -0.29038 | -0.25141 | -0.20592 | -0.16473 |
|  | **Contrastive**  **post-focus** | -0.10104 | -0.14688 | -0.19388 | -0.23782 | -0.27871 | -0.31432 | -0.34445 | -0.36946 | -0.38999 | -0.40777 |
|  |  | -0.42289 | -0.43467 | -0.44254 | -0.44362 | -0.43514 | -0.41588 | -0.38461 | -0.34258 | -0.29242 | -0.2501 |
|  | **Narrow**  **pre-focus** | -0.1554 | -0.2055 | -0.25662 | -0.3021 | -0.3434 | -0.37804 | -0.40542 | -0.42638 | -0.44204 | -0.45409 |
|  |  | -0.46348 | -0.46949 | -0.47147 | -0.46729 | -0.45502 | -0.43263 | -0.40025 | -0.35796 | -0.30834 | -0.26547 |
|  | **Narrow**  **on-focus** | -0.1955 | -0.25283 | -0.30375 | -0.3415 | -0.36765 | -0.38381 | -0.39231 | -0.39527 | -0.39539 | -0.39342 |
|  |  | -0.38916 | -0.38303 | -0.37465 | -0.3633 | -0.34752 | -0.32521 | -0.29452 | -0.25533 | -0.20707 | -0.1612 |
|  | **Narrow**  **post-focus** | -0.09182 | -0.13994 | -0.19 | -0.23651 | -0.28056 | -0.31877 | -0.35135 | -0.37789 | -0.39908 | -0.41673 |
|  |  | -0.4316 | -0.44325 | -0.45067 | -0.45085 | -0.44156 | -0.42114 | -0.38907 | -0.34579 | -0.29383 | -0.25075 |
| **Fortis** | **Baseline** | 0.176556 | 0.163975 | 0.157123 | 0.159272 | 0.172967 | 0.194638 | 0.221233 | 0.249216 | 0.277774 | 0.304936 |
|  |  | 0.329297 | 0.350717 | 0.369121 | 0.384344 | 0.394884 | 0.399547 | 0.396589 | 0.383937 | 0.361243 | 0.332708 |
|  | **Contrastive**  **pre-focus** | 0.139461 | 0.133974 | 0.127942 | 0.122891 | 0.120089 | 0.119524 | 0.12147 | 0.124458 | 0.128965 | 0.134307 |
|  |  | 0.140212 | 0.145856 | 0.151319 | 0.156585 | 0.160834 | 0.163215 | 0.164246 | 0.162482 | 0.158187 | 0.153601 |
|  | **Contrastive**  **on-focus** | 0.256911 | 0.274385 | 0.29239 | 0.313639 | 0.338698 | 0.36689 | 0.397417 | 0.429161 | 0.46111 | 0.491071 |
|  |  | 0.518088 | 0.541767 | 0.562378 | 0.579266 | 0.590879 | 0.594087 | 0.587095 | 0.566717 | 0.531229 | 0.487315 |
|  | **Contrastive**  **post-focus** | 0.122148 | 0.114863 | 0.106322 | 0.09755 | 0.090271 | 0.084549 | 0.081816 | 0.080384 | 0.082383 | 0.085604 |
|  |  | 0.089827 | 0.094311 | 0.098974 | 0.103991 | 0.108869 | 0.113995 | 0.119009 | 0.124904 | 0.130767 | 0.1334 |
|  | **Narrow**  **pre-focus** | 0.109457 | 0.104479 | 0.099506 | 0.096664 | 0.096392 | 0.098939 | 0.103763 | 0.110912 | 0.119871 | 0.129364 |
|  |  | 0.139706 | 0.149333 | 0.158471 | 0.16621 | 0.172042 | 0.175581 | 0.176176 | 0.173489 | 0.167331 | 0.159683 |
|  | **Narrow**  **on-focus** | 0.236279 | 0.250853 | 0.267239 | 0.286876 | 0.310384 | 0.335993 | 0.362796 | 0.391037 | 0.420768 | 0.450168 |
|  |  | 0.47819 | 0.504337 | 0.528299 | 0.548934 | 0.564689 | 0.572204 | 0.569119 | 0.551449 | 0.518735 | 0.472167 |
|  | **Narrow**  **post-focus** | 0.118283 | 0.112436 | 0.105568 | 0.099322 | 0.094606 | 0.09195 | 0.092336 | 0.093772 | 0.096303 | 0.099637 |
|  |  | 0.10337 | 0.106796 | 0.109887 | 0.112437 | 0.114883 | 0.117427 | 0.119977 | 0.121826 | 0.122833 | 0.123732 |

IV**B. Japanese monosyllables**

| **Stop** | **Focus** | **Normalized Time Points** | | | | | | | | | |
| --- | --- | --- | --- | --- | --- | --- | --- | --- | --- | --- | --- |
|  |  | **1/11** | **2/12** | **3/13** | **4/14** | **5/15** | **6/16** | **7/17** | **8/18** | **9/19** | **10/20** |
| **Voiced** | **Baseline** | 0.015315 | -0.03469 | -0.07773 | -0.0958 | -0.09462 | -0.07101 | -0.03461 | 0.009718 | 0.056544 | 0.102282 |
|  |  | 0.144771 | 0.183808 | 0.221096 | 0.255322 | 0.284054 | 0.306214 | 0.31766 | 0.319151 | 0.308694 | 0.289852 |
|  | **Contrastive**  **pre-focus** | -0.10591 | -0.1268 | -0.14199 | -0.14584 | -0.14393 | -0.13683 | -0.1274 | -0.11661 | -0.106 | -0.09558 |
|  |  | -0.0878 | -0.0825 | -0.07901 | -0.07814 | -0.07802 | -0.07699 | -0.07354 | -0.069 | -0.06518 | -0.06362 |
|  | **Contrastive**  **on-focus** | 0.334132 | 0.380132 | 0.42946 | 0.488994 | 0.55568 | 0.628423 | 0.705393 | 0.782237 | 0.856423 | 0.924923 |
|  |  | 0.986218 | 1.040955 | 1.091806 | 1.137669 | 1.171795 | 1.19034 | 1.177564 | 1.126977 | 1.042517 | 0.945374 |
|  | **Contrastive**  **post-focus** | -0.25303 | -0.31021 | -0.36606 | -0.41398 | -0.45559 | -0.49059 | -0.51751 | -0.54042 | -0.55756 | -0.57184 |
|  |  | -0.58341 | -0.5935 | -0.60002 | -0.60104 | -0.59326 | -0.573 | -0.54186 | -0.49833 | -0.44573 | -0.39717 |
|  | **Narrow**  **pre-focus** | -0.07382 | -0.09067 | -0.10263 | -0.10517 | -0.10133 | -0.09442 | -0.08457 | -0.07375 | -0.06336 | -0.05402 |
|  |  | -0.04634 | -0.0412 | -0.0384 | -0.0383 | -0.03997 | -0.04308 | -0.04754 | -0.04899 | -0.04459 | -0.03743 |
|  | **Narrow**  **on-focus** | 0.269691 | 0.325875 | 0.381568 | 0.443364 | 0.506086 | 0.572061 | 0.640851 | 0.709865 | 0.776896 | 0.839423 |
|  |  | 0.89721 | 0.949603 | 0.994528 | 1.031279 | 1.055431 | 1.060157 | 1.039578 | 0.990428 | 0.910896 | 0.821652 |
|  | **Narrow**  **post-focus** | -0.20896 | -0.28407 | -0.35703 | -0.41961 | -0.4726 | -0.51474 | -0.55036 | -0.57885 | -0.60254 | -0.62125 |
|  |  | -0.63657 | -0.64983 | -0.65976 | -0.66592 | -0.66551 | -0.65508 | -0.63302 | -0.59442 | -0.54274 | -0.48778 |
| **Voiceless** | **Baseline** | 0.647268 | 0.681415 | 0.711566 | 0.737372 | 0.757399 | 0.77263 | 0.782801 | 0.790562 | 0.794957 | 0.797947 |
|  |  | 0.799308 | 0.796461 | 0.788474 | 0.772419 | 0.747942 | 0.71332 | 0.667437 | 0.613505 | 0.558225 | 0.514742 |
|  | **Contrastive**  **pre-focus** | 0.311212 | 0.335021 | 0.35473 | 0.366818 | 0.372541 | 0.371088 | 0.36497 | 0.353283 | 0.338348 | 0.320324 |
|  |  | 0.299024 | 0.274923 | 0.248493 | 0.219566 | 0.191062 | 0.163107 | 0.138842 | 0.118445 | 0.105031 | 0.100934 |
|  | **Contrastive**  **on-focus** | 1.06381 | 1.183029 | 1.290945 | 1.382675 | 1.458156 | 1.515059 | 1.556514 | 1.586251 | 1.60529 | 1.616287 |
|  |  | 1.621027 | 1.619657 | 1.610947 | 1.59544 | 1.565781 | 1.516953 | 1.450373 | 1.359756 | 1.251956 | 1.147386 |
|  | **Contrastive**  **post-focus** | -0.01565 | -0.04575 | -0.07809 | -0.11255 | -0.14779 | -0.18386 | -0.2183 | -0.25357 | -0.28693 | -0.31848 |
|  |  | -0.34857 | -0.3741 | -0.39496 | -0.41037 | -0.4177 | -0.41847 | -0.40772 | -0.38831 | -0.36402 | -0.34443 |
|  | **Narrow**  **pre-focus** | 0.306109 | 0.333786 | 0.354268 | 0.365777 | 0.367642 | 0.364473 | 0.355856 | 0.343275 | 0.326913 | 0.307659 |
|  |  | 0.285811 | 0.261866 | 0.236091 | 0.209768 | 0.182433 | 0.154256 | 0.126228 | 0.101409 | 0.083057 | 0.076083 |
|  | **Narrow**  **on-focus** | 0.94319 | 1.069292 | 1.18113 | 1.274692 | 1.345833 | 1.399081 | 1.439474 | 1.469872 | 1.491779 | 1.503781 |
|  |  | 1.505121 | 1.499333 | 1.484218 | 1.459148 | 1.420959 | 1.36675 | 1.290406 | 1.19463 | 1.086242 | 0.998003 |
|  | **Narrow**  **post-focus** | 0.028485 | 3.15E-05 | -0.0303 | -0.0624 | -0.09463 | -0.12705 | -0.15833 | -0.18985 | -0.22017 | -0.25008 |
|  |  | -0.27825 | -0.30398 | -0.32754 | -0.34765 | -0.36185 | -0.36842 | -0.36424 | -0.34632 | -0.31816 | -0.28695 |

IV**C. Korean disyllables**

| **Stop** | **Focus** | **Normalized Time Points** | | | | | | | | | |
| --- | --- | --- | --- | --- | --- | --- | --- | --- | --- | --- | --- |
|  |  | **1/11** | **2/12** | **3/13** | **4/14** | **5/15** | **6/16** | **7/17** | **8/18** | **9/19** | **10/20** |
| **Aspirated** | **Baseline** | 0.354208 | 0.376924 | 0.398761 | 0.417366 | 0.431614 | 0.443314 | 0.452377 | 0.458242 | 0.462284 | 0.464844 |
|  |  | 0.46524 | 0.464984 | 0.464365 | 0.463714 | 0.462909 | 0.462012 | 0.461236 | 0.460417 | 0.45979 | 0.458924 |
|  | **Contrastive**  **pre-focus** | 0.213532 | 0.22215 | 0.230135 | 0.237769 | 0.243497 | 0.248816 | 0.252788 | 0.256124 | 0.258263 | 0.259068 |
|  |  | 0.25853 | 0.256612 | 0.254166 | 0.250696 | 0.246599 | 0.241964 | 0.236931 | 0.231442 | 0.225916 | 0.220738 |
|  | **Contrastive**  **on-focus** | 0.404005 | 0.443582 | 0.480239 | 0.512012 | 0.537338 | 0.558695 | 0.576305 | 0.590709 | 0.60219 | 0.609718 |
|  |  | 0.615134 | 0.619092 | 0.621956 | 0.623734 | 0.625026 | 0.626535 | 0.628235 | 0.629699 | 0.631474 | 0.63279 |
|  | **Contrastive**  **post-focus** | 0.157745 | 0.16547 | 0.172518 | 0.179145 | 0.183142 | 0.186156 | 0.18839 | 0.188941 | 0.1885 | 0.186741 |
|  |  | 0.183369 | 0.178849 | 0.173277 | 0.166763 | 0.159678 | 0.151561 | 0.143186 | 0.134381 | 0.125908 | 0.117692 |
|  | **Narrow**  **pre-focus** | 0.172363 | 0.186328 | 0.199933 | 0.212114 | 0.221827 | 0.229903 | 0.236344 | 0.240881 | 0.243047 | 0.243325 |
|  |  | 0.242134 | 0.239932 | 0.236932 | 0.232933 | 0.228575 | 0.223536 | 0.218376 | 0.213108 | 0.20797 | 0.20325 |
|  | **Narrow**  **on-focus** | 0.40627 | 0.444239 | 0.478923 | 0.508047 | 0.531073 | 0.549418 | 0.564773 | 0.576708 | 0.585791 | 0.592663 |
|  |  | 0.597098 | 0.599638 | 0.601122 | 0.601687 | 0.601666 | 0.601199 | 0.600503 | 0.59965 | 0.598996 | 0.598305 |
|  | **Narrow**  **post-focus** | 0.138617 | 0.14972 | 0.16009 | 0.167478 | 0.172604 | 0.175681 | 0.177475 | 0.177187 | 0.175189 | 0.172612 |
|  |  | 0.169073 | 0.16468 | 0.159674 | 0.154277 | 0.148199 | 0.141707 | 0.135042 | 0.128266 | 0.121938 | 0.116074 |
| **Lenis** | **Baseline** | -0.23018 | -0.29841 | -0.36664 | -0.42728 | -0.47742 | -0.51925 | -0.55005 | -0.57335 | -0.59079 | -0.60367 |
|  |  | -0.61322 | -0.62018 | -0.62412 | -0.62524 | -0.62392 | -0.62049 | -0.61419 | -0.6057 | -0.59452 | -0.58416 |
|  | **Contrastive**  **pre-focus** | -0.27287 | -0.3205 | -0.36918 | -0.41395 | -0.45484 | -0.49179 | -0.52229 | -0.54864 | -0.56966 | -0.58703 |
|  |  | -0.60185 | -0.61449 | -0.62499 | -0.63341 | -0.6403 | -0.64574 | -0.64951 | -0.65214 | -0.65339 | -0.65363 |
|  | **Contrastive**  **on-focus** | -0.29032 | -0.34662 | -0.40334 | -0.45195 | -0.49321 | -0.52618 | -0.55066 | -0.56833 | -0.57922 | -0.58615 |
|  |  | -0.58953 | -0.59063 | -0.58947 | -0.58632 | -0.58117 | -0.57416 | -0.56457 | -0.55237 | -0.53871 | -0.52517 |
|  | **Contrastive**  **post-focus** | -0.18906 | -0.23865 | -0.29 | -0.33984 | -0.38655 | -0.42962 | -0.46762 | -0.50018 | -0.52897 | -0.55404 |
|  |  | -0.57555 | -0.59449 | -0.611 | -0.62501 | -0.63695 | -0.64658 | -0.65433 | -0.66002 | -0.66382 | -0.66577 |
|  | **Narrow**  **pre-focus** | -0.26346 | -0.30724 | -0.35296 | -0.39619 | -0.43645 | -0.47293 | -0.50486 | -0.53213 | -0.55324 | -0.57052 |
|  |  | -0.58439 | -0.59536 | -0.60382 | -0.61092 | -0.61597 | -0.61965 | -0.62217 | -0.62341 | -0.62407 | -0.62387 |
|  | **Narrow**  **on-focus** | -0.33023 | -0.37883 | -0.42664 | -0.46813 | -0.5009 | -0.52621 | -0.54416 | -0.55718 | -0.5655 | -0.5703 |
|  |  | -0.57282 | -0.5729 | -0.57126 | -0.56803 | -0.56287 | -0.55587 | -0.54671 | -0.53546 | -0.52205 | -0.5089 |
|  | **Narrow**  **post-focus** | -0.25078 | -0.28495 | -0.32137 | -0.35861 | -0.39532 | -0.43086 | -0.46304 | -0.49303 | -0.51835 | -0.541 |
|  |  | -0.56075 | -0.5784 | -0.59397 | -0.60766 | -0.61915 | -0.62863 | -0.6359 | -0.64116 | -0.64413 | -0.64479 |
| **Fortis** | **Baseline** | 0.040509 | 0.015267 | -0.00618 | -0.01639 | -0.01257 | 0.003341 | 0.031575 | 0.062816 | 0.095488 | 0.126766 |
|  |  | 0.157128 | 0.185593 | 0.211516 | 0.234618 | 0.255058 | 0.273162 | 0.288904 | 0.302374 | 0.314043 | 0.323675 |
|  | **Contrastive**  **pre-focus** | 0.028897 | 0.027388 | 0.024994 | 0.023267 | 0.022026 | 0.023792 | 0.026058 | 0.030238 | 0.035273 | 0.040487 |
|  |  | 0.045738 | 0.051147 | 0.056067 | 0.061029 | 0.065687 | 0.070355 | 0.075538 | 0.080762 | 0.086301 | 0.090979 |
|  | **Contrastive**  **on-focus** | 0.028161 | 0.03141 | 0.036078 | 0.044456 | 0.058517 | 0.078074 | 0.101048 | 0.128835 | 0.16034 | 0.19488 |
|  |  | 0.230261 | 0.265012 | 0.298388 | 0.329367 | 0.357542 | 0.382849 | 0.405291 | 0.42528 | 0.442656 | 0.457296 |
|  | **Contrastive**  **post-focus** | -0.00522 | -0.01354 | -0.02311 | -0.03165 | -0.03961 | -0.04641 | -0.05086 | -0.05412 | -0.05463 | -0.05362 |
|  |  | -0.05237 | -0.05051 | -0.04875 | -0.04709 | -0.04573 | -0.04467 | -0.04404 | -0.04357 | -0.04328 | -0.04294 |
|  | **Narrow**  **pre-focus** | 0.043396 | 0.040827 | 0.040862 | 0.043598 | 0.049025 | 0.057104 | 0.0665 | 0.075673 | 0.082952 | 0.089511 |
|  |  | 0.095976 | 0.101821 | 0.106894 | 0.111339 | 0.114867 | 0.117537 | 0.119326 | 0.120402 | 0.120776 | 0.120629 |
|  | **Narrow**  **on-focus** | 0.077431 | 0.087862 | 0.10029 | 0.11552 | 0.134876 | 0.156298 | 0.17905 | 0.203092 | 0.228776 | 0.255148 |
|  |  | 0.282024 | 0.308748 | 0.334628 | 0.358122 | 0.379106 | 0.39728 | 0.413362 | 0.427915 | 0.440764 | 0.452368 |
|  | **Narrow**  **post-focus** | -0.05647 | -0.07296 | -0.09018 | -0.10329 | -0.11481 | -0.12122 | -0.12563 | -0.12681 | -0.12582 | -0.12394 |
|  |  | -0.12119 | -0.11763 | -0.11367 | -0.10906 | -0.10418 | -0.09907 | -0.09392 | -0.08921 | -0.08492 | -0.0815 |

IV**D: Japanese disyllabic targets**

| **Stop** | **Focus** | **Normalized Time Points** | | | | | | | | | |
| --- | --- | --- | --- | --- | --- | --- | --- | --- | --- | --- | --- |
|  |  | **1/11** | **2/12** | **3/13** | **4/14** | **5/15** | **6/16** | **7/17** | **8/18** | **9/19** | **10/20** |
| **HL-Voiced** | **Baseline** | 0.181149 | 0.140691 | 0.099801 | 0.076357 | 0.06498 | 0.064039 | 0.081145 | 0.107361 | 0.144343 | 0.185647 |
|  |  | 0.228519 | 0.268451 | 0.30726 | 0.344192 | 0.375012 | 0.401933 | 0.419871 | 0.432835 | 0.438642 | 0.43476 |
|  | **Contrastive**  **pre-focus** | 0.003225 | -0.01968 | -0.04419 | -0.06245 | -0.07289 | -0.08123 | -0.08262 | -0.07801 | -0.0703 | -0.06098 |
|  |  | -0.04952 | -0.038 | -0.03127 | -0.02969 | -0.03404 | -0.04476 | -0.06375 | -0.08946 | -0.12088 | -0.14676 |
|  | **Contrastive**  **on-focus** | 0.304875 | 0.339411 | 0.372736 | 0.412883 | 0.458272 | 0.507008 | 0.561409 | 0.61656 | 0.673012 | 0.728065 |
|  |  | 0.779817 | 0.831464 | 0.877476 | 0.924006 | 0.968279 | 1.006451 | 1.040235 | 1.067724 | 1.086536 | 1.098265 |
|  | **Contrastive**  **post-focus** | -0.29378 | -0.34809 | -0.40488 | -0.45744 | -0.50713 | -0.55201 | -0.5923 | -0.62963 | -0.65777 | -0.68387 |
|  |  | -0.70712 | -0.72781 | -0.74586 | -0.765 | -0.78385 | -0.80363 | -0.82544 | -0.84802 | -0.87083 | -0.88603 |
|  | **Narrow**  **pre-focus** | -0.15078 | -0.16217 | -0.17484 | -0.1799 | -0.18026 | -0.17484 | -0.16264 | -0.1479 | -0.12683 | -0.10573 |
|  |  | -0.0867 | -0.06822 | -0.04968 | -0.03468 | -0.0227 | -0.01348 | -0.00947 | -0.0098 | -0.01471 | -0.01583 |
|  | **Narrow**  **on-focus** | 0.221631 | 0.24392 | 0.264948 | 0.294426 | 0.326864 | 0.363875 | 0.409033 | 0.45549 | 0.507193 | 0.565526 |
|  |  | 0.620789 | 0.674112 | 0.725244 | 0.771733 | 0.813909 | 0.850566 | 0.879073 | 0.902354 | 0.917327 | 0.919396 |
|  | **Narrow**  **post-focus** | -0.39122 | -0.42965 | -0.46911 | -0.50499 | -0.53586 | -0.56511 | -0.58787 | -0.60801 | -0.62609 | -0.64018 |
|  |  | -0.65427 | -0.66984 | -0.68656 | -0.70418 | -0.72312 | -0.74359 | -0.76527 | -0.78786 | -0.80982 | -0.82422 |
| **HL-Voiceless** | **Baseline** | 0.503589 | 0.53801 | 0.573204 | 0.608472 | 0.639664 | 0.667539 | 0.694051 | 0.717961 | 0.735559 | 0.749864 |
|  |  | 0.760395 | 0.765316 | 0.764968 | 0.759921 | 0.751133 | 0.737572 | 0.719585 | 0.697467 | 0.675075 | 0.65791 |
|  | **Contrastive**  **pre-focus** | 0.299129 | 0.309409 | 0.318683 | 0.325153 | 0.327246 | 0.327108 | 0.32272 | 0.315028 | 0.304954 | 0.291648 |
|  |  | 0.276787 | 0.258273 | 0.239006 | 0.219952 | 0.19706 | 0.1711 | 0.143371 | 0.113406 | 0.086757 | 0.068597 |
|  | **Contrastive**  **on-focus** | 0.868106 | 0.948938 | 1.027559 | 1.100227 | 1.161637 | 1.215661 | 1.264762 | 1.305697 | 1.337663 | 1.364102 |
|  |  | 1.38445 | 1.39791 | 1.407967 | 1.413385 | 1.414213 | 1.411396 | 1.405122 | 1.395196 | 1.384221 | 1.37773 |
|  | **Contrastive**  **post-focus** | -0.08692 | -0.12893 | -0.17253 | -0.21409 | -0.25561 | -0.29716 | -0.33576 | -0.37444 | -0.40858 | -0.44143 |
|  |  | -0.47511 | -0.50287 | -0.53014 | -0.55836 | -0.58519 | -0.61158 | -0.6381 | -0.66266 | -0.68272 | -0.69491 |
|  | **Narrow**  **pre-focus** | 0.360433 | 0.373227 | 0.385264 | 0.395039 | 0.401978 | 0.40584 | 0.407772 | 0.406387 | 0.402922 | 0.394936 |
|  |  | 0.385413 | 0.372855 | 0.357329 | 0.337989 | 0.315965 | 0.29208 | 0.265919 | 0.239238 | 0.214272 | 0.194485 |
|  | **Narrow**  **on-focus** | 0.816784 | 0.895925 | 0.972815 | 1.045107 | 1.104858 | 1.159603 | 1.207744 | 1.245247 | 1.275637 | 1.298917 |
|  |  | 1.312167 | 1.320622 | 1.325212 | 1.326634 | 1.323977 | 1.315647 | 1.304009 | 1.288506 | 1.274277 | 1.26795 |
|  | **Narrow**  **post-focus** | -0.03542 | -0.06526 | -0.09676 | -0.12772 | -0.15985 | -0.1938 | -0.22442 | -0.25618 | -0.28956 | -0.31867 |
|  |  | -0.34759 | -0.37965 | -0.41308 | -0.44586 | -0.47911 | -0.51378 | -0.54776 | -0.57964 | -0.60441 | -0.62137 |
| **LH-Voiced** | **Baseline** | -0.72551 | -0.84652 | -0.97071 | -1.0748 | -1.16777 | -1.24553 | -1.30475 | -1.34721 | -1.37662 | -1.39385 |
|  |  | -1.40343 | -1.40292 | -1.3885 | -1.34758 | -1.28029 | -1.18792 | -1.06892 | -0.92562 | -0.77276 | -0.66422 |
|  | **Contrastive**  **pre-focus** | -0.60389 | -0.7295 | -0.85949 | -0.97563 | -1.07624 | -1.16481 | -1.23375 | -1.28653 | -1.32307 | -1.34285 |
|  |  | -1.35163 | -1.34821 | -1.33068 | -1.29718 | -1.2394 | -1.16346 | -1.06876 | -0.95586 | -0.83751 | -0.74473 |
|  | **Contrastive**  **on-focus** | -0.47208 | -0.57539 | -0.68136 | -0.77547 | -0.85504 | -0.92629 | -0.98103 | -1.02361 | -1.04948 | -1.06336 |
|  |  | -1.06433 | -1.04652 | -1.00467 | -0.94278 | -0.84868 | -0.72393 | -0.57104 | -0.39219 | -0.20351 | -0.06909 |
|  | **Contrastive**  **post-focus** | -0.48993 | -0.60687 | -0.728 | -0.84021 | -0.94041 | -1.03234 | -1.10916 | -1.17168 | -1.2254 | -1.26182 |
|  |  | -1.28578 | -1.30137 | -1.30783 | -1.30252 | -1.28587 | -1.25921 | -1.22387 | -1.17901 | -1.13497 | -1.10502 |
|  | **Narrow**  **pre-focus** | -0.57254 | -0.68826 | -0.80725 | -0.91787 | -1.01052 | -1.09648 | -1.16968 | -1.22519 | -1.26882 | -1.30249 |
|  |  | -1.31291 | -1.30978 | -1.29017 | -1.25139 | -1.19315 | -1.11403 | -1.01396 | -0.89521 | -0.77092 | -0.68865 |
|  | **Narrow**  **on-focus** | -0.5531 | -0.6616 | -0.77281 | -0.86843 | -0.95064 | -1.02129 | -1.07361 | -1.1127 | -1.13748 | -1.15144 |
|  |  | -1.15451 | -1.1444 | -1.11252 | -1.05727 | -0.9701 | -0.85253 | -0.70559 | -0.53254 | -0.34954 | -0.22191 |
|  | **Narrow**  **post-focus** | -0.69381 | -0.79584 | -0.90049 | -0.99659 | -1.08274 | -1.15991 | -1.22567 | -1.28055 | -1.32224 | -1.3558 |
|  |  | -1.37999 | -1.39617 | -1.4002 | -1.39385 | -1.3754 | -1.34495 | -1.30296 | -1.24947 | -1.19595 | -1.1568 |
| **LH-Voiceless** | **Baseline** | -0.49539 | -0.58191 | -0.67034 | -0.75723 | -0.83245 | -0.90152 | -0.96222 | -1.01425 | -1.05494 | -1.08433 |
|  |  | -1.10141 | -1.10338 | -1.09305 | -1.07296 | -1.04019 | -0.99629 | -0.94195 | -0.8809 | -0.82311 | -0.78293 |
|  | **Contrastive**  **pre-focus** | -0.71445 | -0.76947 | -0.82602 | -0.884 | -0.93766 | -0.9874 | -1.03292 | -1.07252 | -1.10264 | -1.12477 |
|  |  | -1.13902 | -1.14014 | -1.132 | -1.1154 | -1.09129 | -1.05902 | -1.02165 | -0.97971 | -0.94584 | -0.92153 |
|  | **Contrastive**  **on-focus** | -0.39399 | -0.43705 | -0.48122 | -0.52606 | -0.56965 | -0.60383 | -0.63296 | -0.65871 | -0.67593 | -0.68326 |
|  |  | -0.67903 | -0.66542 | -0.63832 | -0.59741 | -0.54453 | -0.4772 | -0.40224 | -0.32623 | -0.26486 | -0.22468 |
|  | **Contrastive**  **post-focus** | -0.53235 | -0.62157 | -0.71188 | -0.79884 | -0.87726 | -0.95282 | -1.02527 | -1.08356 | -1.13244 | -0.53235 |
|  |  | -1.20733 | -1.22551 | -1.23444 | -1.23737 | -1.23487 | -1.22339 | -1.20618 | -1.18657 | -1.17241 | -1.20733 |
|  | **Narrow**  **pre-focus** | -0.42481 | -0.50248 | -0.58211 | -0.6616 | -0.73493 | -0.79979 | -0.85827 | -0.90834 | -0.95065 | -0.98359 |
|  |  | -1.0042 | -1.01472 | -1.01427 | -1.00248 | -0.98031 | -0.95164 | -0.91174 | -0.86273 | -0.81704 | -0.78793 |
|  | **Narrow**  **on-focus** | -0.55202 | -0.60284 | -0.65476 | -0.70594 | -0.75308 | -0.79641 | -0.83141 | -0.86083 | -0.88173 | -0.89323 |
|  |  | -0.8944 | -0.88284 | -0.85631 | -0.81839 | -0.76775 | -0.70518 | -0.63485 | -0.55365 | -0.47813 | -0.42736 |
|  | **Narrow**  **post-focus** | -0.46208 | -0.5349 | -0.60964 | -0.68549 | -0.75725 | -0.82393 | -0.88765 | -0.94663 | -0.99837 | -1.04053 |
|  |  | -1.07342 | -1.09861 | -1.11744 | -1.12634 | -1.12916 | -1.12723 | -1.11847 | -1.1054 | -1.09228 | -1.08279 |

Appendix V. Statistics of significance region

VA. Japanese syllables across focus conditions

*Significantly different regions across different discourse conditions (HL) (Marginally significant difference based on pointwise critical values is marked within parentheses)*

|  | **Narrow**  **pre-focus vs. baseline** | **Narrow**  **on-focus vs. baseline** | **Narrow**  **post-focus vs. baseline** | **Contrastive**  **pre-focus vs. baseline** | **Contrastive**  **on-focus vs. baseline** | **Contrastive**  **post-focus vs. baseline** |
| --- | --- | --- | --- | --- | --- | --- |
| Voiceless Stops | not sig. | (1.98%~16.83%; 31.68%~42.57%; 45.54%~60.40%; 67.33%~84.16%) | 0∼100% | (0~4.95%; 8.91%~9.9%; 30.69%~33.66%; 39.6%~56.44%; 66.34%~73.27%; 98.02%~100%) | 65.35%~68.32%; 98.02%~99.01% (0~65.35%; 68.32%~98.02%; 99.01%~100%) | 0∼100% |
| Voiced Stops | 0∼100% | 0∼100% | 0∼100% | 0∼100% | 20.79%~100% (15.84%~20.79%) | 0∼100% |

*Significantly different regions across different discourse conditions (LH) (Marginally significant differences are marked in parentheses)*

|  | **Narrow pre-focus**  **vs. baseline** | **Narrow on-focus**  **vs. baseline** | **Narrow post-focus**  **vs. baseline** | **Contrastive pre-focus**  **vs. baseline** | **Contrastive on-focus**  **vs. baseline** | **Contrastive post-focus**  **vs. baseline** |
| --- | --- | --- | --- | --- | --- | --- |
| Voiceless Stops | not sig. | not sig. | not sig. | not sig. | (7.92%~27.72%; 34.65%~95.05%) | not sig. |
| Voiced Stops | not sig. | not sig. | not sig. | not sig. | (16.83%~29.7%; 52.48%~67.33%; 89.11%~95.05%) | not sig. |

VB. Korean syllables across stop types

*Significantly different regions of three stop pairs in seven discourse conditions for monosyllabic targets (Marginally significant differences are marked with parentheses)*

|  | **Baseline** | **Narrow**  **pre-focus** | **Narrow**  **on-focus** | **Narrow**  **post-focus** | **Contrastive**  **pre-focus** | **Contrastive**  **on-focus** | **Contrastive**  **post-focus** |
| --- | --- | --- | --- | --- | --- | --- | --- |
| Fortis vs. lenis stops | (4.95%~7.92%; 12.87%~14.85%; 32.67%~96.04%) | (81.19%~92.08%) | (2.97%~98.02%) | (47.52%~50.5%) | (74.26%~84.16%) | (0~100%) | Not sig. |
| Lenis vs. aspirated stops | 9.9%~93.07% (0~9.9%; 93.07%~100%) | (2.97%~21.78%; 29.7%~96.04%) | 1.98%~97.03% (0~1.98%; 97.03%~100%) | (5.94%~94.06%) | (3.96%~95.05%) | 0~98.02% (98.02%~100%) | (14.85%~90.1%) |
| Aspirated vs. fortis stops | Not sig. | Not sig. | Not sig. | Not sig. | Not sig. | Not sig. | Not sig. |

*Significantly different regions of three stop pairs in seven discourse conditions for disyllabic targets (Korean) (Marginally significant differences are marked with parentheses)*

|  | **Baseline** | **Narrow**  **pre-focus** | **Narrow**  **on-focus** | **Narrow**  **post-focus** | **Contrastive**  **pre-focus** | **Contrastive**  **on-focus** | **Contrastive**  **post-focus** |
| --- | --- | --- | --- | --- | --- | --- | --- |
| Fortis vs. lenis stops | (8.91%~94.06%) | (3.96%~25.74%; 42.57%~73.26%; 87.13%~91.09%) | 8.91%~93.07% (0~8.91%; 93.07%~98) | Not sig. | (38.61%~66.34%; 75.25%~86.14%) | 71.29%~86.14% (0~71.29%; 86.14%~100%) | (52.48%~55.45%; 60.4%~61.39%) |
| Lenis vs. aspirated stops | 0%~100% | (0~98.02%) | 0~100% | (10.89%~88.12%) | (14.85%~96.04%) | 0~100% | (0~34.65%; 70.3%~97.03%) |
| Aspirated vs. fortis stops | Not sig. | Not sig. | Not sig. | Not sig. | Not sig. | Not sig. | Not sig. |

Appendix VI. Statistics of post-hoc analysis regarding F0 perturbation magnitude

VIA. Korean monosyllables

Post-hoc analysis of formula: FBDiff~ focus * stop + (1|subject) + (1|item)

(FBDiff = mean f0 of the same target in focus conditions – mean f0 of the same target in baseline)

| **comparing pairs** | **degree of freedom** | **t. ratio** | **p value** |
| --- | --- | --- | --- |
| ASP cf vs. ASP cpf  ASP cf vs. ASP cpsf  ASP nf vs. ASP npf  ASP nf vs. ASP npsf | 2671  2671  2671  2671 | 13.83  14.48  14.04  14.65 | <0.001***  <0.001***  <0.001***  <0.001*** |
| LEN cf vs. LEN cpf  LEN cf vs. LEN cpsf  LEN nf vs. LEN npf  LEN nf vs. LEN npsf | 2671  2671  2671  2671 | 1.87  0.17  1.27  0.35 | 0.92  1.00  1.00  1.00 |
| FOR cf vs. FOR cpf  FOR cf vs. FOR cpsf  FOR nf vs. FOR npf  FOR nf vs. FOR npsf | 2671  2671  2671  2671 | 11.14  12.23  10.46  11.43 | <0.001***  <0.001***  <0.001***  <0.001*** |
| ASP cf vs. FOR cf | 216 | 0.48 | 1.00 |
| ASP cpf vs. FOR cpf | 216 | -1.86 | 0.92 |
| ASP cpsf vs. FOR cpsf  ASP nf vs. FOR nf  ASP npf vs. FOR npf  ASP npsf vs. FOR npsf | 216  216  216  216 | -1.49  1.71  -1.40  -1.09 | 0.99  0.96  1.00  1.00 |
| FOR cf vs. LEN cf  FOR cpf vs. LEN cpf  FOR cpsf vs. LEN cpsf  FOR nf vs. LEN nf  FOR npf vs. LEN npf  FOR npsf vs. LEN npsf | 216  216  216  216  216  216 | 3.86  -4.18  -6.59  3.19  -4.79  -6.41 | 0.02*  0.005**  <0.001***  0.13  <0.001***  <0.001*** |
| LEN cf vs. ASP cf  LEN cpf vs. ASP cpf  LEN cpsf vs. ASP cpsf  LEN nf vs. ASP nf  LEN npf vs. ASP npf  LEN npsf vs. ASP npsf | 216  216  216  216  216  216 | 4.34  -6.04  -8.08  4.90  -6.18  -7.50 | 0.002 **  <0.001***  <0.001***  <0.001***  <0.001***  <0.001*** |

*ASP: aspirated stop; LEN: lenis stop; FOR: fortis stop*

*cf: contrastive on-focus; cpf: contrastive pre-focus; cpsf: contrastive post-focus; nf: narrow on-focus; npf: narrow pre-focus; npsf: narrow post-focus*

*Signif. codes: 0 ‘***’ 0.001 ‘**’ 0.01 ‘*’ 0.05 ‘.’ 0.1 ‘ ’ 1*

VIB. Korean disyllables

Post-hoc analysis of formula: FBDiff~ focus * stop + (1|subject) + (1|item)

(FBDiff = mean f0 of the same target in focus conditions – mean f0 of the same target in baseline)

| **comparing pairs** | **degree of freedom** | **t. ratio** | **p value** |
| --- | --- | --- | --- |
| ASP cf vs. ASP cpf  ASP cf vs. ASP cpsf  ASP nf vs. ASP npf  ASP nf vs. ASP npsf | 962  962  962  962 | 9.16  11.19  8.87  10.70 | <0.001***  <0.001***  <0.001***  <0.001*** |
| LEN cf vs. LEN cpf  LEN cf vs. LEN cpsf  LEN nf vs. LEN npf  LEN nf vs. LEN npsf | 962  962  962  962 | 0.47  -0.27  0.61  -0.31 | 1.00  1.00  1.00  1.00 |
| FOR cf vs. FOR cpf  FOR cf vs. FOR cpsf  FOR nf vs. FOR npf  FOR nf vs. FOR npsf | 962  962  962  962 | 4.05  6.50  4.27  8.73 | 0.007**  <0.001***  0.003**  <0.001*** |
| ASP cf vs. FOR cf | 278 | 2.30 | 0.68 |
| ASP cpf vs. FOR cpf | 278 | -2.71 | 0.38 |
| ASP cpsf vs. FOR cpsf  ASP nf vs. FOR nf  ASP npf vs. FOR npf  ASP npsf vs. FOR npsf | 278  278  278  278 | -2.29  0.73  -3.78  -1.20 | 0.69  1.00  0.02*  1.00 |
| FOR cf vs. LEN cf  FOR cpf vs. LEN cpf  FOR cpsf vs. LEN cpsf  FOR nf vs. LEN nf  FOR npf vs. LEN npf  FOR npsf vs. LEN npsf | 278  278  278  278  278  278 | 1.62  -1.89  -5.02  2.64  -1.49  -6.23 | 0.98  0.91  <0.001***  0.43  0.99  <0.001*** |
| LEN cf vs. ASP cf  LEN cpf vs. ASP cpf  LEN cpsf vs. ASP cpsf  LEN nf vs. ASP nf  LEN npf vs. ASP npf  LEN npsf vs. ASP npsf | 278  278  278  278  278  278 | 3.92  -4.59  -7.31  3.37  -5.27  -7.43 | 0.01 *  <0.001***  <0.001***  0.08.  <0.001***  <0.001*** |

*ASP: aspirated stop; LEN: lenis stop; FOR: fortis stop*

*cf: contrastive on-focus; cpf: contrastive pre-focus; cpsf: contrastive post-focus; nf: narrow on-focus; npf: narrow pre-focus; npsf: narrow post-focus*

*Signif. codes: 0 ‘***’ 0.001 ‘**’ 0.01 ‘*’ 0.05 ‘.’ 0.1 ‘ ’ 1*

VIC. Japanese monosyllables

Post-hoc analysis of formula: FBDiff~ focus * stop + (1|subject) + (1|item)

(FBDiff = mean f0 of the same target in focus conditions – mean f0 of the same target in baseline)

| **comparing pairs** | **degree of freedom** | **t. ratio** | **p value** |
| --- | --- | --- | --- |
| VED cf vs. VED cpf  VED cf vs. VED cpsf  VED nf vs. VED npf  VED nf vs. VED npsf | 1384  1384  1384  1384 | 11.91  16.87  10.47  16.54 | <0.001***  <0.001***  <0.001***  <0.001*** |
| VLE cf vs. VLE cpf  VLE cf vs. VLE cpsf  VLE nf vs. VLE npf  VLE nf vs. VLE npsf | 1384  1384  1384  1384 | 16.25  23.00  14.15  20.10 | <0.001***  <0.001***  <0.001***  <0.001*** |
| VED cf vs. VLE cf | 165 | -0.53 | 1.00 |
| VED cpf vs. VLE cpf | 165 | 3.35 | 0.045* |
| VED cpsf vs. VLE cpsf  VED nf vs. VLE nf  VED npf vs. VLE npf  VED npsf vs. VLE npsf | 165  165  165  165 | 4.95  0.52  3.81  3.70 | <0.001***  1.00  0.01*  0.01* |

*VED: voiced stop; VLE: voiceless stop*

*cf: contrastive on-focus; cpf: contrastive pre-focus; cpsf: contrastive post-focus; nf: narrow on-focus; npf: narrow pre-focus; npsf: narrow post-focus*

*Signif. codes: 0 ‘***’ 0.001 ‘**’ 0.01 ‘*’ 0.05 ‘.’ 0.1 ‘ ’ 1*

VID. Japanese disyllables

Post-hoc analysis of formula: FBDiff~ focus * pitch accent + (1|subject) + (1|item)

(FBDiff = mean f0 of the same target in focus conditions – mean f0 of the same target in baseline)

| **comparing pairs** | **degree of freedom** | **t. ratio** | **p value** |
| --- | --- | --- | --- |
| HL cf vs. LH cf | 273 | 3.04 | 0.10 |
| HL cpf vs. LH cpf | 273 | -4.95 | <0.001*** |
| HL cpsf vs. LH cpsf  HL nf vs. LH nf  HL npf vs. LH npf  HL npsf vs. LH npsf | 273  273  273  273 | -11.66  2.90  -5.00  -11.06 | <0.001***  0.15  <0.001***  <0.001*** |

*cf: contrastive on-focus; cpf: contrastive pre-focus; cpsf: contrastive post-focus; nf: narrow on-focus; npf: narrow pre-focus; npsf: narrow post-focus*

*Signif. codes: 0 ‘***’ 0.001 ‘**’ 0.01 ‘*’ 0.05 ‘.’ 0.1 ‘ ’ 1*
